# A pressure-tolerant, miniature ocean-sensing tag with acoustic telemetry for real-time CTD monitoring

**DOI:** 10.1126/sciadv.aee9809

**Published:** 2026-08-19

**Authors:** Shao-Hao Lu, Jun Lu, Yi Li, Minsu Park, Jaeyoung Yoo, Aljon Salalila, Zhiqun Daniel Deng, Xueju Sophie Wang

**Affiliations:** ^1^ Department of Materials Science and Engineering, University of Connecticut, Storrs, CT 06269, USA.; ^2^ Energy and Environment Directorate, Pacific Northwest National Laboratory, Richland, WA 99354, USA.; ^3^ Querrey Simpson Institute for Bioelectronics, Northwestern University, Evanston, IL 60208, USA.; ^4^ Department of Naval Architecture and Marine Engineering, University of Michigan, Ann Arbor, MI 48109, USA.; ^5^ Institute of Materials Science, University of Connecticut, Storrs, CT 06269, USA.

## Abstract

Real-time ocean monitoring enables instantaneous oceanographic analysis and supports maritime operations. However, conventional sensors require metallic housings to withstand high hydrostatic pressure, increasing their weight and power demand. Soft electronics offer a promising alternative, but their functionality and commonly used electromagnetic communication are ineffective underwater. Here, we present a pressure-tolerant, miniaturized ocean-sensing tag that seamlessly integrates soft conductivity, temperature, and depth (CTD) sensors with acoustic transducers for in situ monitoring and underwater data transmission. Measuring 17 mm by 20 mm by 7.5 mm and weighing 4.90 g, the tag maintains reliable performance under hydrostatic pressures of up to 15 MPa, corresponding to a depth of approximately 1,500 m, and achieves underwater transmission ranges of up to 492.7 m. Field testing in the Salish Sea demonstrates wireless, real-time CTD monitoring with measurement accuracy comparable to commercial sensors and reliable acoustic telemetry (87.34% detection efficiency and 100% accuracy over evaluated transmission distances). This ocean sensing platform enables energy-efficient deployment on diverse platforms and significantly advances next-generation ocean observation and deep-sea exploration.

## INTRODUCTION

Real-time monitoring of the ocean’s physical, chemical, and biological properties enables instantaneous oceanographic analysis and timely decision-making in maritime operations such as commercial shipping, fishing, and naval activities (*1*–*3*). Conductivity, temperature, and depth (CTD) measurements are particularly critical, as they provide fundamental data for understanding ocean biogeochemistry, determining seawater density (*4*, *5*), and supporting both ocean current modeling (*6*) and weather forecasting (*7*, *8*). To collect CTD data globally, CTD profilers have been deployed on various ocean observation platforms for over a decade, including profiling floats (*7*, *9*), rosette samplers (*10*, *11*), and autonomous underwater vehicles (AUVs) (*12*–*14*). However, existing CTD devices face several challenges for real-time monitoring and deep-sea investigation (table S1), including bulky and heavy metallic protective housings required to withstand high hydrostatic pressure, limited underwater communication capabilities, and high energy consumption. For example, high-depth-rated commercial CTD sensors can operate at depths of up to 10,500 m (*15*, *16*), but they exceed 30 cm in length, and their titanium housings increase their weight to over 1.5 kg (*15*, *16*). This form factor in turn increases the size and power demand of sampling systems and impedes their integration with compact, energy-efficient platforms.

To broaden deployment options and expand the coverage of CTD-sampled regions, a downsized CTD-satellite relay data logger (SRDL) system has been developed and deployed on marine animals as mobile sampling platforms (*1*). Despite its reduced size (10.5 cm by 7 cm by 4 cm), the use of this profiler remains limited to large, air-breathing species such as seals and sea turtles due to its weight (545 g) and dependence on periodic surfacing for satellite data relay (*17*–*19*). Exploiting ongoing advances in soft electronics (*20*–*23*), researchers have developed a flexible CTD sensing system (Marine Skin) (*24*, *25*), enabling noninvasive biotagging of small fish species, such as seabream. Although this device has been tested under lab-simulated conditions, including high hydrostatic pressure and exposure to Red Sea water (28 days), its capacity for real-time CTD monitoring has not been thoroughly assessed (*24*). Specifically, continuous CTD recordings have been evaluated exclusively in an 80 cm-deep water tank (*25*). Moreover, the system relies on Bluetooth for wireless communication, which becomes ineffective underwater due to the rapid attenuation of electromagnetic signals, allowing data transmission only when the animal surfaces (*25*).

In this work, we have developed a miniature, pressure-tolerant ocean-sensing tag that integrates (1) soft CTD sensors for operation in harsh environments, (2) a battery-powered, lightweight printed circuit board (PCB) housing the electronics, (3) underwater acoustic data transmission, and (4) a wearable elastomeric casing for noninvasive biotagging. We first demonstrated the reliability of these soft CTD sensors through comprehensive lab-controlled evaluations in artificial seawater, including dynamic bending, cyclic pressurization, and long-term pressure tolerance tests (15 MPa hydrostatic pressure, corresponding to an ocean depth of 1500 m). We then demonstrated the effectiveness of acoustic telemetry, achieving hundred-meter-scale underwater transmission and robust data modulation of ultrasound signals. The system’s capacity for real-time CTD monitoring was further validated through field testing in the Salish Sea, confirming reliable in situ CTD sensing and acoustic data transmission in a marine environment. By integrating soft CTD sensors with acoustic telemetry, our developed ocean-sensing tag reduces the device’s size and weight by two orders of magnitude compared to conventional CTD sensors, while enabling real-time oceanographic measurements and deployment across diverse energy-efficient platforms, including fish biologging and soft robotics (*26*–*30*). Collectively, the capabilities offered by our platform could substantially advance ocean sensing efforts, enhancing timely data access and supporting deep-sea exploration.

## RESULTS

### Design of a soft ocean-sensing tag

**Fig. 1.**
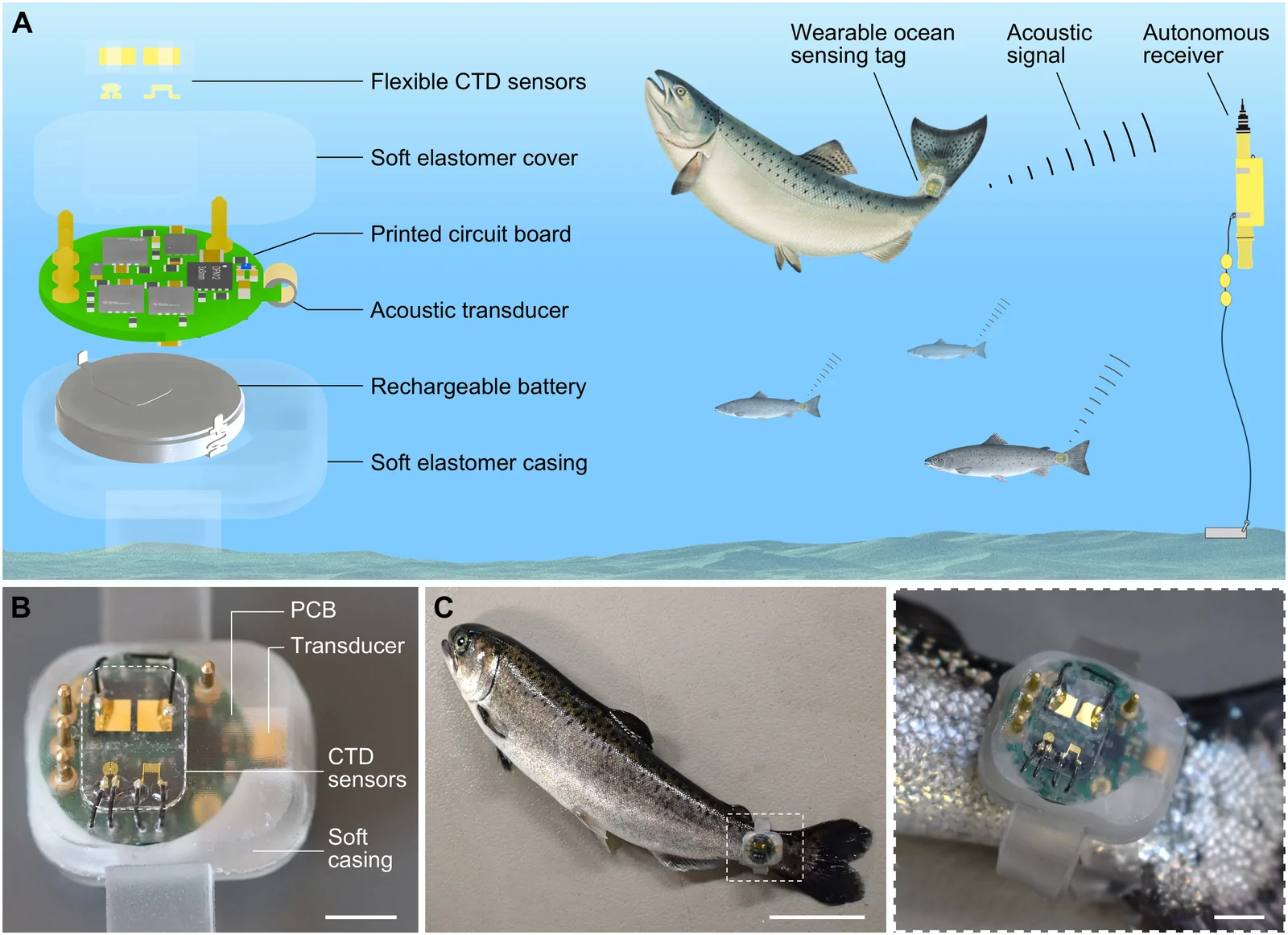
Design and application of a soft ocean-sensing tag. (**A**) Exploded-view schematic of the ocean-sensing tag and its envisioned applications in biotelemetry and real-time ocean monitoring. (**B**) Top-view photograph of the assembled tag. (**C**) Photograph of the tag attached to a juvenile Chinook salmon, with a magnified view of the attached tag near the tail region. Scale bars: 5 mm in (B), 5 cm in the main panel of (C), and 5 mm in the magnified view in (C).

The ocean-sensing tag consists of soft, pressure-tolerant CTD sensors, a miniaturized PCB, an acoustic transducer, a rechargeable battery, and a soft elastomeric casing (Fig. 1A). The millimeter-scale CTD sensors, which measure salinity, temperature, and pressure, utilize photolithographically patterned ultrathin metal films (gold/chromium, 100 nm/10 nm) as sensing elements and are encapsulated in soft polymers (polyimide, 4 μm and parylene C, 5 μm) (fig. S1). To ensure accurate sensing through close contact with seawater, the sensors are mounted on the top surface and connected to the PCB underneath. The PCB integrates power and signal processing circuits that convert CTD measurements into voltage signals and drive the acoustic transducer for underwater data transmission. To achieve waterproofing and reliable operation in marine environments, the PCB, battery, and acoustic transducer are encapsulated in a molded epoxy resin followed by a conformal parylene C coating. The entire assembly is housed in a soft elastomer casing, which comprises a top cover, bottom shell, and watch band, and is designed for wearability and flexibility (fig. S2). The fully assembled miniature ocean-sensing tag measures just 17 mm by 20 mm by 7.5 mm (Fig. 1B). Including its 7.5 mm-wide watch band, the entire device weighs only 4.90 g in air and has a volume of 3.46 cm^3^. Our device achieves a two-order-of-magnitude reduction in both weight and volume relative to the CTD-SRDL systems commonly used in ocean biotelemetry (545 g, ∼250 cm^3^) (table S1) (*31*).

This miniaturization, combined with real-time underwater data transmission, is especially advantageous for tag deployment across diverse animal-borne platforms, ranging from large air-breathing mammals to small fish species. To demonstrate its potential application in biotelemetry, we attached the tag to the tail of a juvenile Chinook salmon (30 cm long, 270 g; Fig. 1C and fig. S3), highlighting its suitability for use on small fish species as mobile CTD sensing platforms. Unlike conventional tagging methods, such as the adhesives employed with seals and sea turtles (*32*, *33*) or the surgical sutures used in fish tagging (*34*), our device enables a noninvasive tagging approach. The tag can be secured or removed within seconds using the adjustable watch band and plastic buckle, greatly reducing handling time and animal stress. To enhance attachment robustness for freely moving animals, an additional layer of underwater epoxy or adhesive could be applied to the watchband while maintaining a minimally disruptive tagging procedure (*32*, *33*). Future designs could further incorporate a replaceable watchband (e.g., a band slot with a release mechanism), enabling deployment across animals of varying sizes and morphologies. Beyond biotelemetry, the compact, lightweight ocean-sensing tag can be integrated into a wide range of platforms, including autonomous profilers, diving equipment, and soft robotics (*29*, *30*).

### Circuit design of the ocean-sensing system

**Fig. 2.**
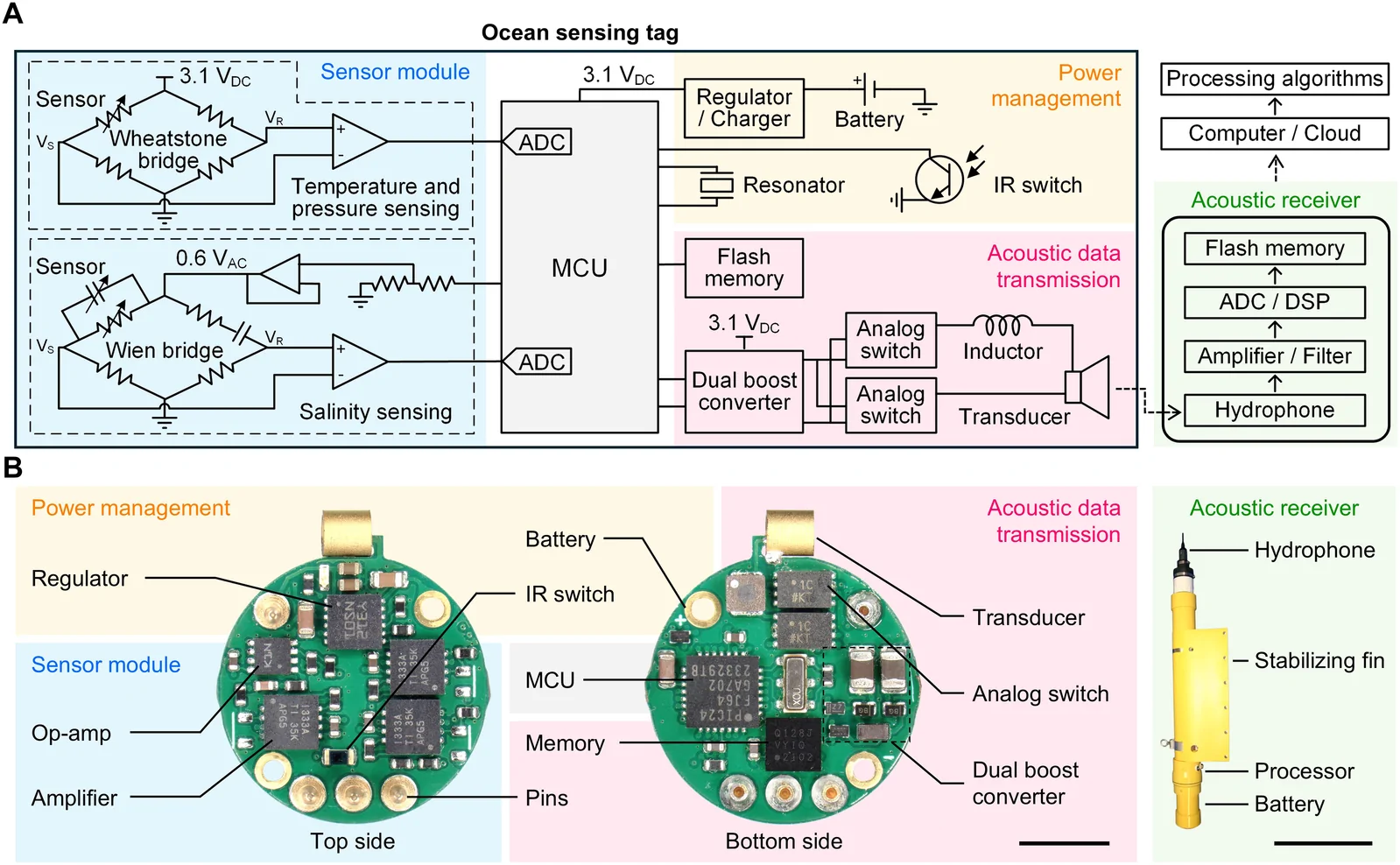
System architecture and device design of the ocean-sensing tag and acoustic receiver. (**A**) Circuit diagram of the ocean-sensing tag and block diagram of acoustic data reception. (**B**) Microscope images of the top and bottom sides of the PCB layout (scale bar: 5 mm), and a photograph of the autonomous acoustic receiver (scale bar: 50 cm).

The circuit design of the ocean-sensing tag is presented in Fig. 2A and note S1. Wheatstone bridges, supplied with 3.1 V_DC_ from a voltage regulator, convert resistive measurements from the temperature and pressure sensors into corresponding voltage signals. Similarly, impedance measurements from the salinity sensor are converted into voltage signals using a Wien bridge, supplied with 0.6 V_AC_ from a buffered voltage divider (*35*). The voltage from each bridge circuit is amplified by an instrumentation amplifier and digitized into 12-bit data by the analog-to-digital converter (ADC) of the microcontroller unit (MCU). The sensor data are transmitted via a lead zirconate titanate (PZT) piezoelectric acoustic transducer driven by high-amplitude alternating voltage (fig. S4). The transmitted acoustic signals are data-encoded ultrasound with a frequency of 416.7 kHz, which is outside the range of typical background noise in marine environments (*36*). The autonomous acoustic receiver captures these signals with a hydrophone, amplifies and filters the resulting electrical signals, and sends them to a digital signal processor (DSP) for decoding. Finally, data-processing algorithms are applied to remove false-positive decodes and extract clean CTD datasets. The raw sensor data are simultaneously stored in the tag’s flash memory and can be retrieved after tag recovery to validate the acoustically transmitted data and serve as a backup data source.

To accommodate the voltage requirements of various components, the tag integrates a rechargeable Li-ion battery with a charger/regulator that provides a constant 3.1 V supply to the MCU and analog circuitry. For salinity sensing in marine environments, a 0.6 V_AC_ excitation is employed to prevent chloride-induced corrosion of the gold electrodes and to avoid polarization effects during ion conductivity measurements, based on our previous study (*37*). The MCU serves as a pulse-width modulation (PWM) generator, producing a 3.1 V pulse signal at 1 kHz with a 50% duty cycle, which is then stepped down to 0.6 V using a buffered voltage divider circuit. For acoustic transducer actuation, a dual boost converter and analog switches produce high-amplitude alternating voltage waveforms with data modulation, reaching peak amplitudes of ±25.5 V. These waveforms are applied to the transducer and inductor, creating LC resonance between the inductor’s inductance and the transducer’s capacitance, thereby enhancing the efficiency of electrical-to-acoustic energy conversion (*38*). To minimize interference between the low-voltage sensing front-end and the high-voltage acoustic driver, the tag utilizes a strict temporal isolation strategy. Sensor acquisition and acoustic transmission are separated by sufficient settling intervals to ensure complete dissipation of residual energy. Specifically, the data sampling interval is set to 1 s. Within each cycle, the sensor module is enabled for 1 ms to allow signal stabilization, acquisition and ADC conversion, after which it is powered down and the acquired data are stored in the MCU. The boost converter is then activated for approximately 50 ms to charge the storage capacitors and generate the required voltage for acoustic transmission (*38*). Following a 744 μs data transmission, the boost converter is turned off for more than 0.9 s before the next sensing cycle, ensuring that residual ringing, thermal transients, and electromagnetic interference from the boost/driver stage have fully dissipated (fig. S5).

The tag incorporates multiple power management strategies to improve energy efficiency. An infrared (IR) switch is used to activate the device prior to deployment, minimizing standby power loss (*36*), while a resonator provides a precise clock for acoustic modulation and task scheduling. Temporal isolation between sensing and transmission further reduces power consumption. Within each 1 s data sampling interval, the MCU briefly enables the instrumentation amplifier (∼1 ms) in the sensing module and the boost converter (∼50 ms) in the acoustic transmission module only during their designated operating periods, after which both are completely turned off to conserve power. Sequential acquisition of data from each sensor requires a total cycle time of 3 s. The device consumes less than 1 μA in the idle mode and up to 40 mA during active sensing and transmission. Under these conditions, a 60 mAh Li-ion battery supports approximately six days of operation, which is suitable for short-term applications such as diving wearables with regular recharging. For longer-term deployments (e.g., biotelemetry), extending the sampling interval (e.g., to ≥1 min) can substantially reduce power consumption, enabling operational lifetimes on the order of months to a year.

The miniaturized PCB (Fig. 2B) has a diameter of 1.5 cm, with the acoustic transducer measuring 2.65 mm in length and 2.54 mm in outer diameter. The inner and outer walls of the acoustic transducer are gold-coated and connected to the PCB. The pins on the PCB serve as electrical interfaces for data retrieval from the onboard flash memory and for battery recharging, enabling reuse of the ocean-sensing tag. These pins remain exposed after epoxy encapsulation and are further coated with an outer Parylene C layer, which protects them from direct seawater exposure and reduces the risk of leakage at the pin-epoxy interface. After tag retrieval from underwater operation, the pins can be accessed for external connection by removing the outer coating. Details of the pin configuration and maintenance strategies are provided in note S2. The autonomous acoustic receiver, measuring 152 cm in length, comprises a hydrophone, battery packs, circuit boards, and PVC housing. It is also equipped with a plastic fin to stabilize its orientation under intense flows (*39*).

### Characterization of CTD sensing performance and reliability

**Fig. 3.**
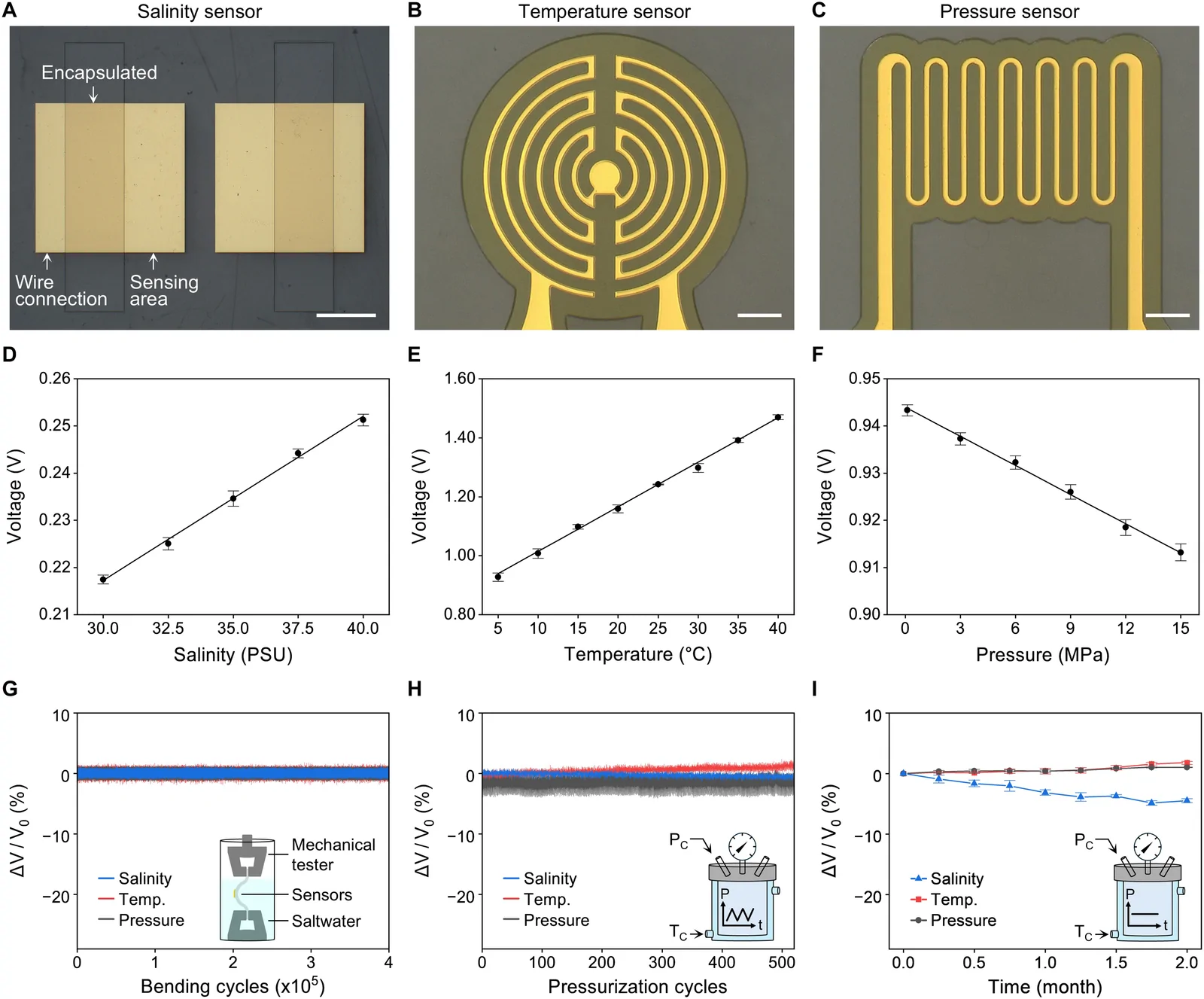
Characterization of the sensing performance and reliability of CTD sensors. Microscope images of (**A**) salinity, (**B**) temperature, and (**C**) pressure sensors. Scale bars: (A) 1 mm, (B, C) 200 μm. Calibration curves of (**D**) salinity, (**E**) temperature, and (**F**) pressure sensors. Reliability tests of the ocean-sensing tag, including (**G**) cyclic dynamic bending, (**H**) cyclic pressurization and depressurization between 0.1 and 15 MPa, and (**I**) long-term pressure tolerance at a constant pressure of 15 MPa.

Microscopic images of our salinity, temperature, and pressure sensors highlight their millimeter-scale dimensions and microstructured designs (Fig. 3, A to C). The salinity sensor has two electrodes with sensing areas exposed for direct contact with seawater. The temperature and pressure sensors are designed with 40-μm-wide serpentine patterns to enhance mechanical stretchability and compliance. Calibration curves (n = 3 per sensor type) show linear relationships between measured voltage and salinity, temperature, and pressure within the tested ranges. The salinity sensor exhibits a linear voltage increase from 30 to 40 practical salinity units (PSU) at a constant temperature of 25°C (Fig. 3D), attributed to increased ionic concentration and electrical conductivity. The temperature sensor demonstrates a linearly increasing voltage from 5 to 40°C (Fig. 3E), consistent with the positive temperature coefficient of resistance of the metallic sensing element (ΔR = TCR × R_0_ × ΔT), where resistance (and thus voltage) rises with temperature (*40*). In contrast, the pressure sensor displays a decreasing voltage as hydrostatic pressure increases from 0.1 to 15 MPa at a constant temperature of 25°C (Fig. 3F), because compressive strain reduces the trace resistance by shortening its conductive path and increasing its cross-sectional area (*41*). The measured sensor sensitivities are 0.003 V/PSU for salinity sensing, 0.015 V/°C for temperature sensing, and −0.002 V/MPa for pressure sensing.

We comprehensively evaluated the reliability of the ocean-sensing tag under simulated marine conditions, including dynamic mechanical deformation, cyclic pressurization, and long-term harsh environment exposure (fig. S6). First, we performed a cyclic bending test on CTD sensors in artificial seawater to assess their mechanical robustness (Fig. 3G). Over 400,000 bending cycles, the relative voltage changes (ΔV/V_0_) of the salinity, temperature, and pressure sensors remained within ±1.48%, ±1.91%, and ±1.63%, respectively, indicating high mechanical resilience. To test durability for repeated vertical profiling, we conducted cyclic pressurization of the ocean-sensing tag between 0.1 and 15 MPa every hour (Fig. 3H; fig. S7), mimicking the pressure change from the sea surface to 1,500 m depth. After 500 cycles, the performance of the salinity and temperature sensors was negligibly affected, with relative output voltage changes (ΔV/V_0_) of −0.570% and +1.064%, respectively. The pressure sensor exhibited negligible hysteresis and high repeatability. Finally, the long-term tolerance test examined the performance consistency of the device in simulated harsh deep-sea environments by subjecting the CTD sensor tag to constant conditions of 25°C, 15 MPa hydrostatic pressure, and 35 PSU salinity over a two-month period (Fig. 3I). All CTD sensors remained functional throughout the duration, exhibiting negligible signal shift. These results collectively validate the high sensitivity, mechanical durability, and long-term stability of the soft CTD sensors and ocean-sensing tag in simulated deep-sea conditions.

### Underwater acoustic telemetry

**Fig. 4.**
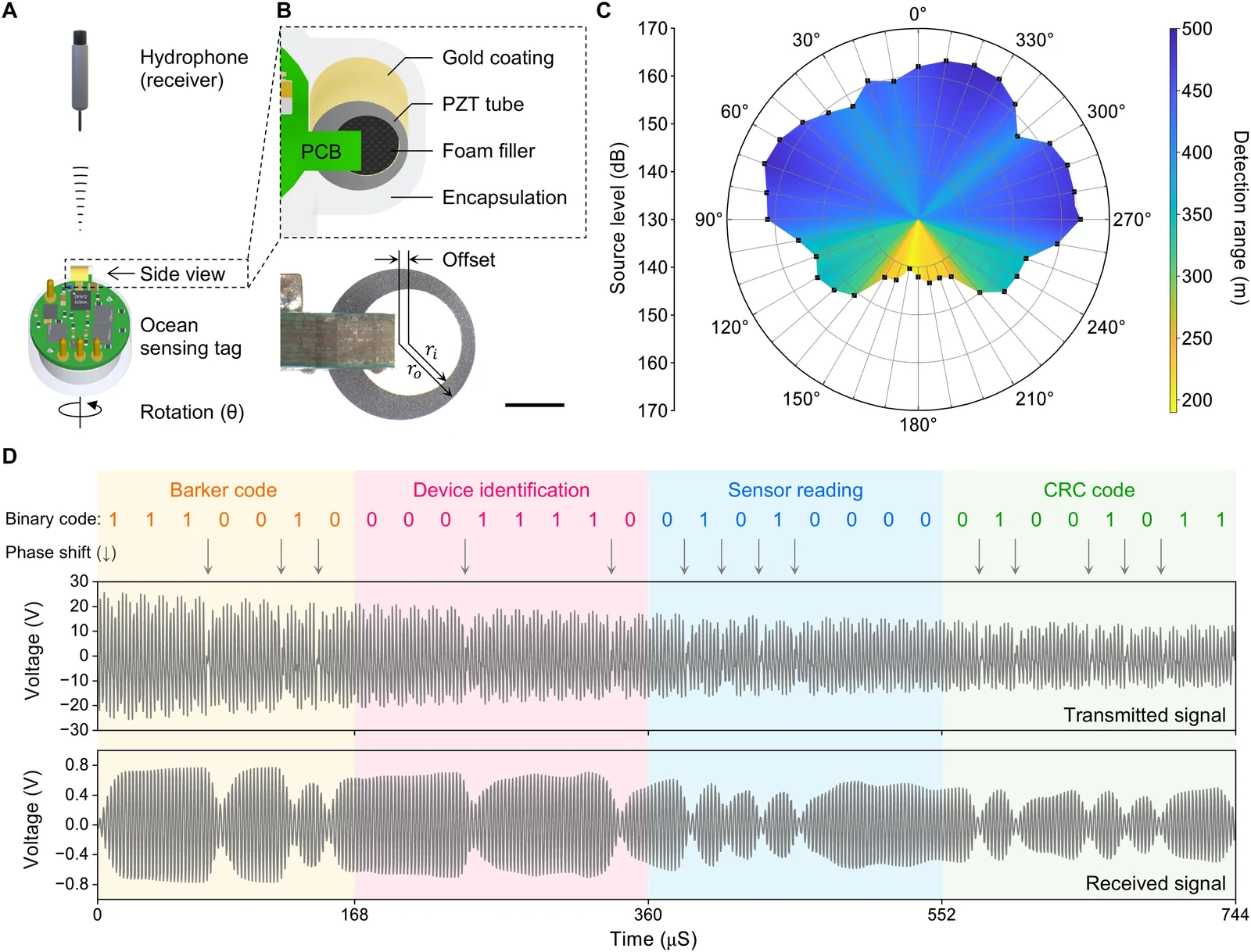
Detection range and data modulation of acoustic telemetry. (**A**) Experimental setup for recording the acoustic signals from a rotating ocean-sensing tag. (**B**) Schematic and microscope image of an acoustic transducer on a PCB, showing the foam filler and inner-wall offset. Scale bar: 1 mm. (**C**) Beam pattern of the acoustic transducer (source level ranging from 130 dB at the center to 170 dB at the outer edge), with color mapping indicating the estimated detection range. (**D**) BPSK-encoded waveforms transmitted from the acoustic transducer and received by the hydrophone, carrying a complete 31-bit data sequence.

To enable real-time underwater data transmission for our ocean-sensing system, we integrated acoustic telemetry with the soft ocean sensors to transmit sensing data via ultrasound, which greatly improves transmission range compared to electromagnetic wireless communication. In order to enhance the transmitted signal strength, the transducer tube was filled with foam backing material (Fig. 4A) to minimize mutual cancellation of ultrasound from the inner and outer surfaces, and its inner wall was offset 0.15 mm from the PCB to direct more acoustic energy toward the front of the tag (Fig. 4B; note S3) (*42*, *43*). The transmission performance was measured by rotating the ocean-sensing tag in an anechoic water tank from 0° to 360° in 10° intervals, with a receiver positioned 1 m away continuously recording the signal strength (source level).

The beam pattern (Fig. 4C) shows the measured source level over a full rotation, reaching a maximum of 164.3 dB at 340°, when the transducer directly faces the receiver, and decreasing to a minimum of 140.5 dB at 170°, when it faces away and the PCB partially obstructs signal transmission. Based on these measurements, along with acoustic attenuation and receiver sensitivity, the effective detection range was estimated assuming propagation at 10°C and a depth of 1 m in either pure water or seawater (35 PSU) (note S4) (*44*–*46*). The maximum detection range reached 492.7 m in pure water (Fig. 4C), demonstrating its applicability in freshwater environments such as rivers, lakes, and dams (*39*, *46*), while in seawater it was reduced to 303.4 m due to increased sound absorption from dissolved salts. Although acoustic propagation depends on water properties (fig. S8), the system maintains a transmission range over hundreds of meters across environmental conditions—nearly two orders of magnitude greater than the few-meter range achievable with electromagnetic waves underwater (*44*, *47*).

To ensure accurate data transmission in noisy marine environments, we employed a modulation technique known as binary phase-shift keying (BPSK). This entails encoding data in ultrasound by altering the phase of the 416.7-kHz carrier wave rather than its amplitude, thereby reducing susceptibility to signal attenuation in seawater (*36*, *48*). Each phase shift in the BPSK waveform represents a 1-to-0 or 0-to-1 transition, enabling the transmission of binary data (note S5; fig. S9). Figure 4D presents a set of 31-bit BPSK-encoded signals transmitted from the acoustic transducer and received by the hydrophone. For purposes of synchronization, the first 7-bit Barker code (1110010) is identical across all encoded signals, allowing receivers to recognize the beginning of a data sequence. The following 8-bit device identification code (00011110) represents the hexadecimal value “1E”, where “1” denotes the tag ID and “E” indicates the pressure sensor. The next 8-bit sensor reading code (01010000) is translated to decimal (80) and then converted into the sensor voltage output (voltage = decimal × 3.1 V / 2^8^). The final 8 bits (01001011) are used for a cyclic redundancy check (CRC), enabling receivers to verify the integrity of the entire data sequence. It should be noted that the number of bits allocated for device identification and sensor reading codes can be adjusted to accommodate different numbers of tags or sensors and to tailor the resolution of the transmitted data. Through BPSK modulation, the received signal preserves distinct phase shifts and maintains data accuracy during underwater propagation, despite substantial amplitude attenuation from ±25.5 V at transmission to ±0.8 V at reception (Fig. 4D). These results demonstrate that acoustic telemetry, combining robust data encoding with an extended detection range, enables effective long-distance data transmission for our ocean sensing system.

### Field testing

**Fig. 5.**
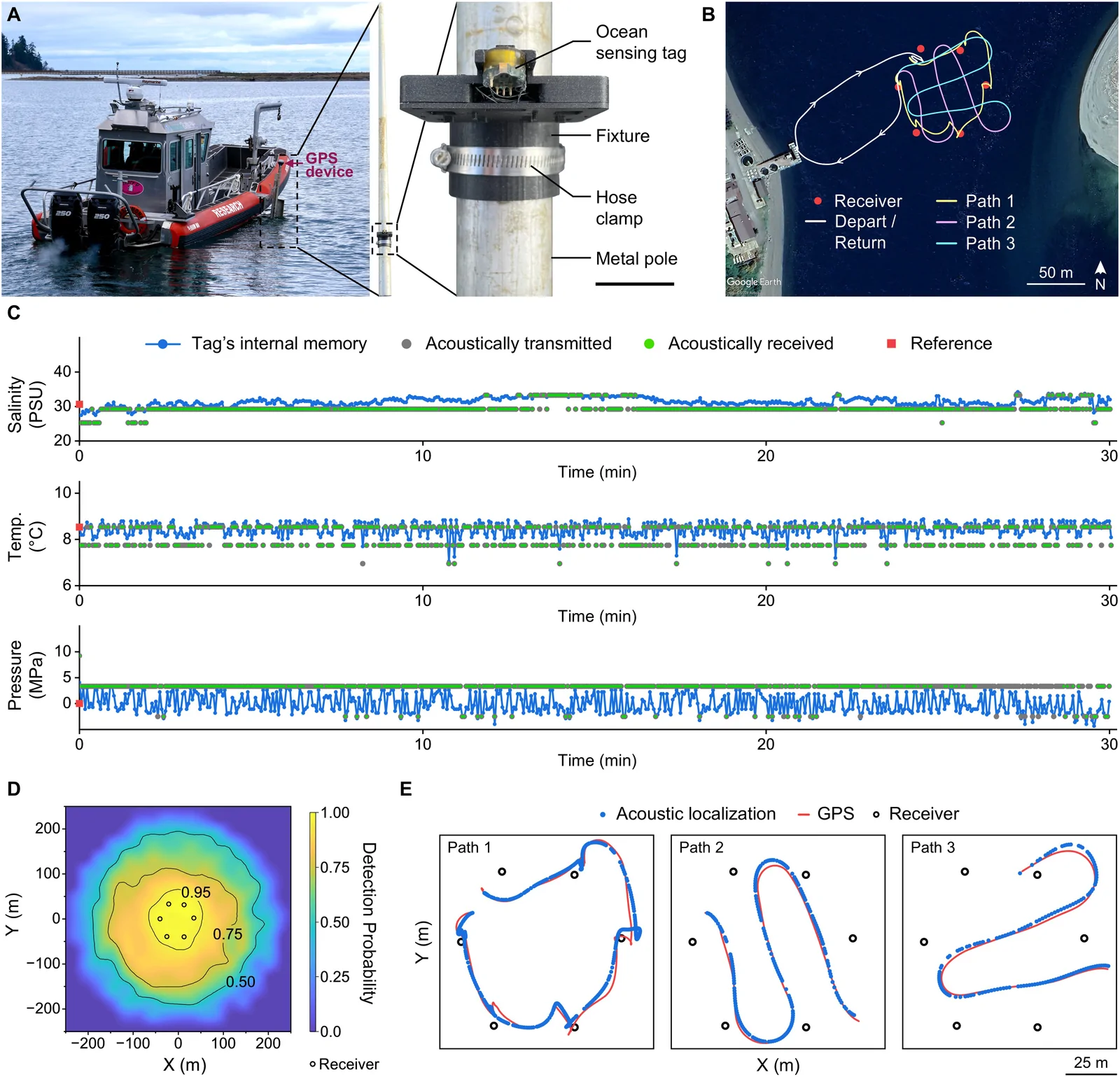
Field testing of the ocean-sensing tag with acoustic telemetry. (**A**) Photograph of field testing in Sequim Bay, Washington, USA, showing an ocean-sensing tag attached to a steel pole and installed on a boat. Scale bar: 5 cm. (**B**) Satellite map indicating boat routes and the locations of deployed receivers. (**C**) Salinity, temperature, and pressure sensor readings obtained from the tag’s internal memory and acoustic telemetry (transmitted and received data), compared with reference values from a commercial CTD sensor. (**D**) Contour plot of detection probability for acoustic transmission by the deployed receiver array. (**E**) Tracking routes derived from underwater acoustic localization and GPS.

The system’s capacity for real-time CTD monitoring was evaluated through field testing in Sequim Bay, Washington, USA, located in the coastal waters of the Salish Sea in the Pacific Ocean (Fig. 5A; fig. S10). For deployment, three ocean-sensing tags, each containing one set of CTD sensors, were mounted to a metal pole using 3D-printed plastic fixtures and hose clamps and submerged at a depth of ∼1.2 m (fig. S11). Six autonomous acoustic receivers were deployed in a hexagonal array and anchored to the seafloor (Fig. 5B), enabling in situ acquisition and decoding of CTD data. For reference, vertical CTD profiles were measured with a commercial instrument (SonTek CastAway-CTD; fig. S12).

Figure 5C presents 30 minutes of continuous CTD measurements recorded during the field trial, including data retrieved from the tag’s internal flash memory, data transmitted and received via acoustic telemetry, and reference values from the commercial CTD. The internally logged measurements yielded average salinity, temperature, and pressure values of 31.369 PSU, 8.464°C, and 0.0115 MPa, respectively. These values closely matched the reference measurements (30.636 PSU, 8.532°C, and 0.0120 MPa) obtained from the vertical profiles at the depth nearest the tag deployment and most representative of the local physical conditions. We used detection efficiency, defined as the ratio of valid detected sensor data to total transmissions, as a metric for assessing acoustic transmission performance (*39*). The overall detection efficiency across all CTD readings was 87.34% across the evaluated transmission distances of up to 100 m; among these valid detections, we observed 100% accuracy of transmitted versus received codes. These results demonstrate the effectiveness of our acoustic telemetry system and confirm successful real-time wireless transmission of CTD data in marine environments. We observed slight discrepancies between the tag’s internally logged (12-bit) and acoustically transmitted (8-bit) data—averaging 0.58%, 0.68% and 4.16% for temperature, pressure, and salinity—due to the lower resolution of the acoustically transmitted data. The resolution of transmitted data can be further improved either by allocating more bits to the sensor readings in the encoding scheme (e.g., a 4-bit device ID with a 12-bit sensor reading), as discussed in the acoustic telemetry section, or by leveraging multiple transmissions (e.g., two full 12-bit sensor readings from three 8-bit encoded acoustic transmissions). In addition, the observed fluctuations in the continuous CTD data are likely caused by waves and boat movement.

To visualize the effective detection area, we summed the detection efficiencies at varying distances from each of the six receivers to calculate the detection probability. This value represents the likelihood that a sensor transmission is received by at least one receiver in the acoustic telemetry system. We plotted the derived detection probability within a 500-m square region as a 2D contour map (Fig. 5D). The receiver array exhibited a high detection probability of 0.95 within a diameter of 125 m. The coverage area extended to 270 m in diameter at a detection probability of 0.75, effectively spanning the distance from the dock to the opposite shore (Klapot Point) and demonstrating the system’s potential for recording CTD data from tagged fish in future biotelemetry studies (fig. S13). Compared with existing CTD devices (table S1), our lightweight, miniature system offers a versatile and effective platform for performing both continuous CTD monitoring and in situ data transmission, with field-evaluated sensing performance comparable to the commercial DST CTD and CTD Biotag systems (table S2) (*49*, *50*).

Furthermore, the tag’s underwater position can be computed from the time of arrival (TOA) of acoustic signals received by multiple receivers. This technique, known as acoustic localization, provides positional information for both recorded CTD data and tagged animals. We compared tag movements obtained by acoustic localization with GPS reference positions as the boat circled the receiver array (Path 1), moved in the north-south direction (Path 2), and then in the east-west direction (Path 3) (Fig. 5E). Across all paths, the tracking efficiency (i.e., the ratio of successfully tracked locations to total transmissions) was 80.20%, offering sufficient localization points to resolve the tag’s trajectories. When we computed the tracking accuracy by quantifying differences between the acoustic localization and GPS, we obtained median absolute errors of 0.90 m (x) and 1.05 m (y), with root mean square (RMS) errors of 1.54 m (x) and 1.71 m (y) (table S3), indicating that the underwater acoustic localization achieves a resolution of approximately 1 m. Based on the above results for sensing, telemetry, and localization, our field-testing data validate the accuracy and reliability of our ocean sensing system under realistic deployment scenarios, confirming the system’s viability for real-time CTD observations.

## DISCUSSION

We have developed a miniaturized, pressure-tolerant ocean-sensing tag with acoustic telemetry for real-time oceanographic measurements. This system offers several advantages over conventional CTD sensors and existing flexible electronic platforms. First, its compact (17 mm × 20 mm × 7.5 mm) and lightweight (4.90 g) design is approximately two orders of magnitude smaller and lighter than commonly used CTD sensors in ocean biotelemetry (e.g., CTD-SRDLs; 545 g, ∼250 cm^3^) (*31*). Second, the soft CTD sensors exhibit stable and consistent performance under high hydrostatic pressure (15 MPa, equivalent to 1,500 m depth) and high salinity (30–40 PSU), significantly extending the applicability of flexible sensors from previously demonstrated low-pressure (∼kPa) (*51*) or low-salinity biofluid environments (*22*) to harsh deep-sea conditions. Third, by leveraging acoustic telemetry, the system achieves an underwater data transmission range of up to 492.7 m, representing a two-order-of-magnitude improvement over electromagnetic communication, such as Bluetooth, used in current flexible electronic systems (*25*). Finally, field testing in the Salish Sea validates the system’s capacity for real-time continuous CTD monitoring and underwater data transmission, demonstrating CTD measurement accuracy comparable to commercial sensors and robust acoustic telemetry performance.

The miniature, lightweight ocean-sensing tag substantially reduces payload and power demands, facilitating integration with diverse energy-efficient sampling platforms. This is exemplified by our demonstration of noninvasive tagging of juvenile salmon as mobile sensing platforms. Furthermore, acoustic localization allows continuous tracking of the tag’s underwater movements, offering spatial context for both animal movements and recorded CTD profiles. Collectively, our ocean-sensing system represents a novel integration of soft electronics and acoustic telemetry, extending the operational domain of flexible electronics into underwater environments and yielding an advanced sensor platform for next-generation ocean exploration.

Building on these demonstrated capabilities, future work can focus on further improving measurement accuracy and deployment scalability. CTD sensor performance may be enhanced by incorporating automatic temperature compensation (ATC) into salinity and pressure measurements to maintain accuracy under varying thermal conditions (*52*). For realistic CTD profiling, sampling strategies could be optimized to reduce noise levels while preserving sufficient temporal resolution to capture vertical gradients. Additionally, although the present pressure sensor operates over a wide range of up to 15 MPa (∼1,500 m depth), its sensitivity at shallow depths could be improved through the use of more responsive capacitive or piezoresistive materials (*53*). Expanded field testing would further evaluate the long-term reliability of the ocean-sensing system in real ocean environments and advance its technology readiness level (TRL) (*54*). Furthermore, developing scalable manufacturing approaches for soft CTD sensors could significantly reduce production costs, providing a cost-effective alternative to commercial CTD sensors that typically cost more than $10,000 (*55*). Such cost reductions would facilitate large-scale global deployment, complementing and extending existing observational networks, including the global Argo network (*56*). The broader deployment of CTD sensors would improve spatiotemporal sampling coverage, strengthening global research on weather patterns and climate systems.

## MATERIALS AND METHODS

### Sensor fabrication

The CTD sensors were fabricated using Au (100 nm)/Cr (10 nm) thin films as sensing components, with soft polymers for substrates and encapsulation, employing fabrication procedures reported in our previous work (*37*, *41*, *57*). The substrate for the salinity sensors was fabricated by depositing a layer of parylene C (5 μm) onto a glass slide via chemical vapor deposition (SCS Labcoter, Specialty Coating Systems). The substrate for the temperature and pressure sensors was fabricated by spin-coating (2,000 rpm, 60 s) the polyimide (PI) precursor onto a pre-coated poly(methyl methacrylate) (PMMA) glass slide. PMMA served as a sacrificial layer for releasing the sensors from the glass. The spin-coated PI was then heated at 130°C for 5 min and cured at 245°C for 70 min in a vacuum oven (YES-58 HMDS Oven, Yield Engineering Systems).

Conductive sensing elements were fabricated by sputtering Au/Cr thin films onto the substrates using a magnetron sputtering system (AJA Orion-8, AJA International), and were patterned into the desired geometries using photolithography and wet etching processes. The patterned salinity-sensing electrodes were encapsulated with a 5-μm top layer of parylene C, and the patterned temperature and pressure sensing traces were encapsulated with a 4-μm top layer of spin-coated PI. To create exposed conductive areas for wire connections and salinity sensing, the top encapsulation layers were patterned using photolithography and reactive ion etching with oxygen plasma. After microfabrication, the salinity sensors were directly released from the glass using a release agent (2% Micro-90 in DI water). The temperature and pressure sensor arrays were removed from the glass by immersing them in acetone for 72 hours to dissolve the PMMA layer, and then transferred to a water-soluble polyvinyl alcohol (PVA) tape. The individual temperature and pressure sensors were separated from the tape by dissolving the PVA in water.

The sequence of integration of CTD sensors onto a soft polydimethylsiloxane (PDMS) substrate follows the steps below to ensure proper attachment and encapsulation, while keeping the salinity-sensing electrodes exposed. The temperature and pressure sensors were first transferred onto the PDMS, soldered to conductive wires, and encapsulated with epoxy at the wire connections. Parylene C was then deposited onto these sensors to maintain their adhesion to the PDMS under mechanical deformation. During the parylene C deposition, the area of the PDMS substrate designated for the salinity sensor was masked with Scotch tape to prevent coating. Afterward, the masking tape was removed, and the salinity sensor was transferred onto the PDMS. Following wire soldering and epoxy encapsulation at the connection sites, semi-cured PDMS was applied around the substrate of the salinity sensor to secure it to the underlying PDMS layer.

### Design and fabrication of the ocean-sensing tag

The circuit for the ocean-sensing tag was designed using Altium Designer software and fabricated by an ISO 9001-certified PCB manufacturer (Bittele Electronics). The firmware for the microcontroller unit (MCU) was programmed using the MPLAB X integrated development environment (IDE) and uploaded to the MCU via the PICkit in-circuit programmer (Microchip Technology) (*36*). The ocean-sensing tag was built with a core MCU and three functional modules for power management, CTD sensing, and acoustic data transmission. Details of the circuit design and electronic components are provided in note S1.

To complete the integration of the ocean-sensing tag, the acoustic transducer was first attached to the PCB using silver-filled conductive epoxy (AA-DUCT 902, Atom Adhesives) instead of soldering to prevent the material from losing its piezoelectric properties at its Curie temperature of 210°C (*36*). An ethylene-propylene-diene monomer closed-cell foam (CCF; McMaster-Carr) was used to fill the transducer to maximize the outward acoustic output. A parylene C layer (25 μm) was deposited onto the assembled PCB and transducer to protect the electrical components from moisture and electrical shorts. The CTD sensors and battery were then soldered to the PCB contact pads that remained uncoated by parylene C using masking materials. The fully integrated ocean-sensing tag was then placed in a silicone mold, encapsulated with insulating epoxy (EPO-TEK 301-2 epoxy resin, Epoxy Technology), cured at 80°C for 3 hours, and subsequently coated with an outer parylene C layer to ensure waterproofing and functionality of the electronics during underwater testing.

The elastomer casing for the wearable device was based on molded PDMS. The molds were fabricated using a 3D printer (Form 3+, Formlabs). A release agent (Ease Release 200, Mann Release Technologies) was applied to the mold surfaces 5 min prior to casting. The PDMS silicone base and curing agent (Sylgard 184, Dow) were mixed at a weight ratio of 10:1. The degassed mixture was then poured into the molds and cured at 80*°*C for 12 hours.

### Tagging of juvenile salmon

The juvenile Chinook salmon (*Oncorhynchus tshawytscha*) used for the tagging demonstration was obtained from the Pacific Northwest National Laboratory (PNNL) Aquatic Research Laboratory (ARL) stock. The juvenile salmon weighed 270 g and measured 30 cm in length. The ocean-sensing tag was attached to the salmon by securing the watch band around its tail and fastening the plastic buckle through the adjustment hole. The tagging process was completed in seconds without the need for anesthesia.

Biotelemetry studies have assessed the effects of tag burden (the ratio of tag weight to fish weight) on the survival, growth, and swimming performance of tagged fish. While some research follows the commonly accepted guideline that the tag burden should remain below 2%, other studies have exceeded this threshold and reported no negative impacts at tag burdens ranging from 6.7 to 8% (*58*–*60*). Our wearable ocean-sensing tag, which imposes only a 1.81% tag burden on juvenile salmon, satisfies these recommended guidelines and is not expected to adversely affect the tagged fish.

### Lab validation

The cyclic bending test was conducted by clamping CTD sensors to the specimen grips of a mechanical tester (UniVert, CellScale), submerging them in artificial seawater, and applying constant displacement. The mechanical tester was programmed to apply the displacement at 1 Hz, generating a curvature radius of 4 mm on the CTD sensors. The artificial seawater was prepared by dissolving sea salt (Instant Ocean, Spectrum Brands) in DI water. The sea salt contains NaCl, MgCl_2_, Na_2_SO_4_, CaCl_2_, KCl, and water of hydration (*61*, *62*). It consists of approximately 80.9 wt% solids and 19.1 wt% water, as determined by evaporation tests at 200°C for 24 h. The salinity of artificial seawater was based on Absolute Salinity (parts per thousand, ppt), defined as the mass ratio of dissolved solids to total seawater mass (g/kg) (*63*). Because Practical Salinity is numerically close to Absolute Salinity, practical salinity units (PSU) were used to express the nominal salinity levels in this study (*64*).

A custom-built pressure vessel system, equipped with a syringe pump and a chiller, was utilized to simulate hydrostatic pressure under deep-sea conditions. The syringe pump (Model 500D, Teledyne ISCO) regulated the hydrostatic pressure by filling the vessel chamber with pressurized DI water. The chiller (Minichiller 300-H OLÉ, Huber) controlled the temperature by circulating water through the jacket surrounding the vessel. To prevent corrosion of the vessel chamber, the ocean-sensing tag was enclosed in a compressible round bellows (inner diameter of ¾ inch, extended length of 1 ft), which was filled with artificial seawater and sealed with rubber stoppers. The bellows was then immersed in DI water inside the pressure vessel, rather than filling the entire vessel with saltwater. During testing, the device was connected to an external USB cable (CAB-12977, SparkFun Electronics) for data collection and powered by an external power supply via wiring routed through the vessel head, as the testing duration (>1 week) exceeded the battery lifetime. The hydrostatic pressure was varied from 0.1 MPa (1 atm at sea level) to 15 MPa (corresponding to a depth of ∼1,500 m). The fully integrated, battery-powered tag was also verified to operate under the same pressurized conditions (fig. S14) and in field deployments, demonstrating its capability for standalone CTD sensing.

Acoustic transmission was tested in an anechoic tank (1.26 m long, 0.95 m wide, and 0.90 m deep) to minimize sub-MHz noise from ultrasound reflections (*44*). The tank was filled with fresh water, and its interior surfaces were lined with a 26-mm-thick anechoic layer (Aptflex F48, Precision Acoustics). The strength (source level) of the transmitted acoustic signals was measured using a hydrophone (Model SC 001-2008-0404, Sonic Concepts). The ocean-sensing tag and hydrophone were positioned 1 m apart and 0.5 m below the water surface. Prior to measurement, the hydrophone was calibrated with an omnidirectional broadband projector hydrophone (Model TC-4034, Reson A/S) (*36*). The beam pattern of the acoustic transmission was measured by rotating the ocean-sensing tag from 0° to 360° in 10° intervals using a motion control unit.

### Field testing

Field testing was conducted at Sequim Bay, Washington, USA, in the coastal waters of the Salish Sea. The tests were performed on February 21 and 22, 2024, during slack tide. Three ocean-sensing tags were attached to a metal pole (length of 3.1 m) using 3D-printed plastic fixtures and hose clamps. The three tags were positioned 15 cm apart and submerged to depths of 1.0–1.3 m. The metal pole was installed into a pole holder, which was then secured to the boat. A survey-grade GPS (REACH RS2+, with centimeter precision) was placed on top of the metal pole to track the movements of the tested devices. Six autonomous acoustic receivers (SR5000, Advanced Telemetry Systems) were deployed 100 m from the floating dock, forming a hexagonal array with a side length of 40 m. Each receiver had positive buoyancy and was anchored to the seafloor using a buoy float line and a steel anchor.

The boat trajectories and receiver locations are displayed on the satellite images in Fig. 5B (Day 1) and fig. S13 (Day 2). On the first day of field testing, the boat first approached the deployed receivers, circled around them (Path 1), moved in the north-south direction within the receiver array (Path 2), then in the east-west direction (Path 3), and finally returned to the floating dock. On the second day of field testing, we additionally drifted the boat 200 m away from the receiver array in both the north and south directions to evaluate the detection range and probability of the acoustic telemetry system within a broader 500-m square region.

During field testing, CTD sensor readings were simultaneously transmitted via acoustic signals and stored in internal flash memory. The data saved in memory were extracted via PCB pins after device recovery and used as raw sensor data. The acoustically transmitted data were received by autonomous receivers, decoded into digital data, and verified through cyclic redundancy checks (CRC) (fig. S15). Each decode was saved along with its time of arrival (TOA), recorded with a precision of 1 μs. These decodes and their TOAs enable filtering algorithms to extract clean CTD datasets and allow the machine learning solver to compute underwater positions of the tag. Specifically, given a nominal pulse repetition interval (PRI) of 1 s for data transmission, the filtering algorithms eliminate false-positive decodes with unexpected transmission timing due to sound reflection, refraction, or signal distortion. First, the multipath filter checks the decodes from each receiver and removes duplicates that occur shortly (<0.3 s) after the initial decode. The initial decode is assumed to be the direct signal from the transmitter, while any identical signal appearing shortly afterward is considered a multipath signal that has reflected off the water surface or sea bottom and taken a longer path (*65*). Next, the PRI filter examines the combined decodes from several receivers and removes those that do not arrive within the predefined PRI time window (*66*). Subsequently, both the memory-saved data and the filtered, acoustically transmitted data were converted to salinity, temperature, and pressure values using the calibration curves obtained with CTD sensors at 8.5°C (fig. S16).

Underwater acoustic localization is based on TOA measurements. Using the TOA of acoustic signals at multiple receivers and the speed of sound in water, the distances from the transmitter to each receiver can be calculated. Given the known positions of the receivers, the location of the transmitter can be determined by solving for the position that best matches the calculated distances to all receivers. We established a neural network to compute the continuous positions of the ocean-sensing tag and effectively track its movements. Further details are provided in note S6.

## Supplementary Materials

### This PDF file includes:

Supplementary Text Figs. S1 to S16 Tables S1 to S3 References
