# Supplementary material for: A pressure-tolerant, miniature ocean-sensing tag with acoustic telemetry for real-time CTD monitoring
Source: Sci Adv. 2026 Aug 19;12(34):eaee9809. doi: 10.1126/sciadv.aee9809 (PMC13488880; doi:10.1126/sciadv.aee9809)
Supplement: Supplementary file 1 [file sciadv.aee9809_sm.pdf]

Science Advances
AAAS

Supplementary Materials for

A pressure-tolerant, miniature ocean-sensing tag with acoustic telemetry for real-time CTD monitoring

Shao-Hao Lu *et al.*

Corresponding author: Zhiqun Daniel Deng, zhiqun.deng@pnnl.gov; Xueju Sophie Wang, xueju.wang@uconn.edu

Sci. Adv. **12**, eaee9809 (2026)
DOI: 10.1126/sciadv.aee9809

This PDF file includes:

Supplementary Text
Figs. S1 to S16
Tables S1 to S3
References

Supplementary Text

Note S1 Circuit design of the ocean sensing tag

The power management module consists of a rechargeable battery, a charger/regulator, an infrared (IR) switch, and a resonator. The batteries used in this study include a 40-mAh pouch cell for wearable devices and a 60-mAh coin cell for lab validation and field testing. The pouch cell (PRT-13852, SparkFun Electronics, USA) measures 9 mm × 18 mm × 2.9 mm. The coin cell (CP 1254 A3, VARTA AG, Germany) has a diameter of 12.1 mm and a height of 5.4 mm. The charger/regulator (STNS01, STMicroelectronics) regulates voltage fluctuations and supplies a constant 3.1 V to power the circuit. It also provides overcharge, over-discharge, and overcurrent protection for the battery. The IR phototransistor switch (Everlight, PT19-21B) is used for wireless programming and device activation prior to deployment. A 10 MHz resonator (CSTNE10M0G520000R0, Murata Electronics) provides a clock reference to precisely control the task timing and the acoustic carrier frequency during data transmission.

The CTD sensing module includes the CTD sensors, bridge circuits, and amplifiers. The Wheatstone bridges, each composed of a resistive temperature or pressure sensor and three reference resistors, are powered by a 3.1 V source. The voltage at the sensing node (V_S) varies with the resistance change of the sensor. The voltage difference between the sensing (V_S) and reference (VR) nodes is amplified by instrumentation amplifiers (INA 333, Texas Instruments, USA), producing amplified voltage outputs that are linearly proportional to physical changes. For salinity sensing, a voltage conditioning circuit consisting of a resistive voltage divider and an Op-amp (TSV632, STMicroelectronics) buffer reduces the potential of the pulse signal from the microcontroller unit (MCU) to 0.6 V_{AC} for salinity sensing. The Wien bridge converts the impedance changes of the salinity sensor into corresponding voltage signals. Similarly, the differential voltages between the two middle nodes of the bridge circuits (V_S and V_R) are amplified by the INA, generating outputs that are linearly proportional to salinity changes. All amplified voltages are fed into the MCU (PIC24FJ64GA702, Microchip Technology) and then subjected to analog-to-digital conversion (ADC) and processed for acoustic transmission.

The acoustic data transmission module consists of a dual boost converter circuit, two analog switches, an inductor, and an acoustic transducer. The dual boost converter, which contains two capacitors, two Schottky diodes, two transistor switches, and a shared inductor1, boosts the 3.1 V input to a higher voltage. The analog switches then produce data-modulated voltage waveforms, which are applied to the transducer and inductor. Electrical resonance between the inductor and the transducer's capacitance enhances the efficiency of conversion of electrical energy into acoustic waves. The acoustic transducer (PZT-5H tube, TRS Technologies Inc., USA), with gold-coated inner and outer walls connected to the PCB, generates radial vibrations in response to applied voltage signals and emits ultrasonic waves for acoustic data transmission.

Note S2 Design and maintenance of PCB pins

The ocean-sensing tag has four PCB pins that serve as the electrical connectors. These four pins correspond to VCC (power), GND (ground), TXD (transmit data), and RXD (receive data). They enable battery recharging and data retrieval from flash memory, thereby supporting reuse of the ocean-sensing tag. The pins are electroless nickel immersion gold (ENIG)-plated, which provides inherent resistance to corrosion in seawater. To extend the longevity and reusability of the pins, cleaning and maintenance between field deployments are important. After each field deployment, including the field testing reported here, the exposed pins were cleaned and dried prior to data offloading and battery charging. For repeated deployments, routine cleaning and inspection for corrosion or discoloration are recommended to preserve connector integrity and long-term system stability (*67*).

Note S3 Design concept of acoustic transducer

The acoustic transducer (PZT tube) converts applied voltage into radial mechanical vibrations, generating ultrasonic signals. To enhance the acoustic output strength, the acoustic transducer is filled with foam material, and the center of its inner wall is offset away from the circuit board. The foam filler serves as an acoustic backing material, attenuating the ultrasound emitted from the inner wall of the transducer and thereby reducing mutual cancellation (*i.e.,* destructive interference) of ultrasound propagating from the inner and outer surfaces. This maximizes the outward transmission of acoustic energy (*42, 43*). The center offset of the acoustic transducer is designed to reduce transmission energy at the rear, where the signal is blocked by the PCB, and to direct more acoustic energy toward the front of the transducer. Based on Equation (S1), the resonance frequency (f_r) of a PZT tube is determined by the frequency constant (N_c) of the PZT, as well as the OD and ID (outer and inner diameter) of the tube (*42*).

$$f_r = \frac{2N_c}{OD + ID} \tag{S1}$$

Thus, the non-concentric design positions the thinnest portion of the transducer wall facing the front of the tag, generating greater displacement and a stronger acoustic signal toward the front of the device without altering the frequency of the ultrasonic signals.

Note S4 Estimation of detection range for acoustic transmission

The detection range of the acoustic transmitter depends on its source level (SL), transmission loss (TL), the background noise level (NL), and the receiver's signal-to-noise ratio (SNR) (*44*):

$$\mathrm{SL} - \mathrm{TL} - \mathrm{NL} - \mathrm{SNR} = 0 \tag{S2}$$

Transmission loss results from a combination of spreading loss due to sound-wave propagation and absorption loss within the water medium:

$$\mathrm{TL} = s log10\,(r) + \alpha r \tag{S3}$$

where *r* represents the distance between the transmitter and receiver, *s* is the spreading factor, and α is the sound absorption coefficient.

The absorption coefficient (α, in dB/km) is a function of the sound frequency (*f*, in kHz), depth (*z*, in km), and water properties, including temperature (*T*, in °C), pH, and salinity (*S*, in parts per thousand, ppt) (*45, 46*). The following equation expresses the sound absorption coefficient in seawater as the sum of contributions from boric acid, magnesium sulphate ($MgSO_4$), and pure water, respectively (*45*):

$$\begin{aligned}\alpha = {} & 0.106\,\frac{f_1 f^2}{f^2 + {f_1}^2}\; e^{(pH-8)/0.56} \\ & + 0.52\left(1 + \frac{T}{43}\right)\left(\frac{S}{35}\right)\frac{f_2 f^2}{f^2 + {f_2}^2}\; e^{-z/6} \\ & + 0.00049\, f^2\, e^{-(T/27 + z/17)}\end{aligned} \tag{S4}$$

where f_1 and f_2 are the relaxation frequencies of boric acid and magnesium sulphate (*45*):

$$f_1 = 0.78\left(\frac{S}{35}\right)^{\frac{1}{2}} e^{T/26} \tag{S5}$$

$$f_2 = 42\, e^{T/17} \tag{S6}$$

Thus, the detection range (*r*) of the acoustic transmitter can be calculated by solving the following equation:

$$\mathrm{SL} - (s log10\,(r) + \alpha r) - (\mathrm{NL} + \mathrm{SNR}) = 0 \tag{S7}$$

where SL is the measured source level. An empirical value of $s = 15$ is considered an accurate estimate for water bodies with depths of several dozen meters (*44*). We assumed a nominal receiver detection threshold of NL + SNR = 95 dB, based on the typical minimum detectable signal strength of the Trident Acoustic Receiver (SR3001, Advanced Telemetry Systems, Inc., Isanti, MN, USA), which is widely used in JSATS applications.

Based on these equations, we estimated the detection range of 416.7 kHz ultrasound signals at pH=8 and near-surface water depth (D=0.001 km) under different water properties. In pure water (10°C, S=0 ppt, α=58.74 dB/km), the maximum source level (164.3 dB) corresponds to a detection range of 492.7 m. In seawater (10°C, S=35 ppt, α=105.79 dB/km), higher sound absorption

reduces the maximum detection range to 303.4 m. To further assess the environmental effects on acoustic telemetry performance, we plotted the absorption coefficient and detection range under different temperature and salinity conditions (Fig. S8). The results indicate that (1) increasing salinity raises the absorption coefficient and reduces the detection range; (2) in pure water, increasing temperature lowers the absorption coefficient and improves the detection range; whereas (3) in seawater, increasing temperature leads to stronger acoustic absorption and shorter detection ranges. Overall, the acoustic telemetry system exhibits a greater detection range in pure water, exceeding 1,000 m at elevated temperatures. In seawater, although dissolved salts increase sound absorption and shorten the detection range, the system still maintains transmission over a hundred-meter scale in marine environments.

Note S5 Data modulation and transmission of acoustic telemetry

Binary data can be encoded and decoded between digital signals and acoustic BPSK signals. Specifically, no phase change in the BPSK waveform represents a 1-to-1 or 0-to-0 transition, while a phase change indicates a 1-to-0 or 0-to-1 transition. Each bit of data requires 10 cycles of the carrier wave (416.7 kHz), resulting in a period of 24 µs per bit. Therefore, the entire 31-bit BPSK signal has a duration of 744 µs (*68*). In addition, the received signal exhibits lower voltage amplitude during phase-shifting because the transducer cannot instantly change the phase of oscillation and requires several cycles to complete the phase shift. This also explains why the BPSK waveform uses 10 cycles to encode a single bit of data (*68*).

The continuous decrease in the amplitude of the transmitted signal within the 31-bit code is mainly due to the energy depletion of the dual 47 µF capacitors in the boost converter (*38*). Despite this, the analysis of the received data indicates that this amplitude decay does not significantly impact the Bit Error Rate (BER) for the trailing segments of the packet. This is due to two factors: 1) Receiver Sensitivity: The Juvenile Salmon Acoustic Telemetry System (JSATS) hydrophone array and digital signal processing (DSP) algorithms utilize an adaptive thresholding technique that tracks the envelope of the incoming signal, maintaining reliable symbol synchronization even as the amplitude decreases across the packet (*68*); 2) Energy Reservation: The total energy stored in the dual-capacitance bank was specifically sized to ensure that the final bit of the 31-bit ID still exceeds the minimum detectable source level and remains within 3 dB of the initial bit, ensuring consistent decoding performance across all 31 bits without a statistically significant increase in bit errors at the end of the sequence.

Furthermore, the acoustic telemetry system supports simultaneous deployment of multiple tags by minimizing signal collisions through a short packet duration and asynchronous transmission. Because each 31-bit encoded acoustic signal lasts only 744 µs (0.744 ms), even microsecond-scale differences in transmission timing can create temporal separation that greatly reduces packet overlap. With a nominal Pulse Rate Interval (PRI) of 1 s, slight clock variation and differences in underwater propagation distance generate natural timing offsets among tags. As a result, even if

transient overlap occurs, the signals drift apart in subsequent cycles, preventing persistent collisions and maintaining high detection efficiency under dense deployment conditions.

Note S6 Underwater acoustic localization

Acoustic localization is achieved by using the time of arrival (TOA) of tag codes received by multiple receivers to calculate the position of the sound source. After data filtering, the tag codes from all deployed receivers are combined to solve the tag locations using a physics-informed machine learning solver, which includes a forward propagation process and a decoder built with prior information. For each transmitted tag signal, the forward propagation process first transforms the time of arrival at the i-th receiver ($t_{a,i}$) into high-dimensional Fourier features, which are then fed into a fully-connected neural network (FCNN) to output raw positions (X, Y, Z). These raw positions are subsequently mapped to physical positions (x, y, z) using the decoder. Next, the physical positions $\mathbf{x} = (x, y, z)$ and time of pin (t_p) are used to calculate the time residual (ε_i) in the following physical equation of signal travel time:

$$t_{a,i} - t_p + \varepsilon_i = \frac{|\mathbf{x} - \mathbf{x}_{r,i}|}{c} \tag{S8}$$

where $|\mathbf{x} - \mathbf{x}_{r,i}|$ is the distance between the tag and the i-th receivers, and c is the signal propagation speed. Assuming the detection time errors follow a Gaussian distribution, we can then evaluate ε_i and calculate its probability. By applying forward propagation to the signals received by all receivers, the probabilities of all ε_i can be obtained. A gradient descent method is then employed to minimize the negative log-likelihood loss function with respect to the network's trainable parameters until a convergence criterion is met. Continuous computation provides a series of localization points and creates a tracking trajectory, enabling the monitoring of tag movements.

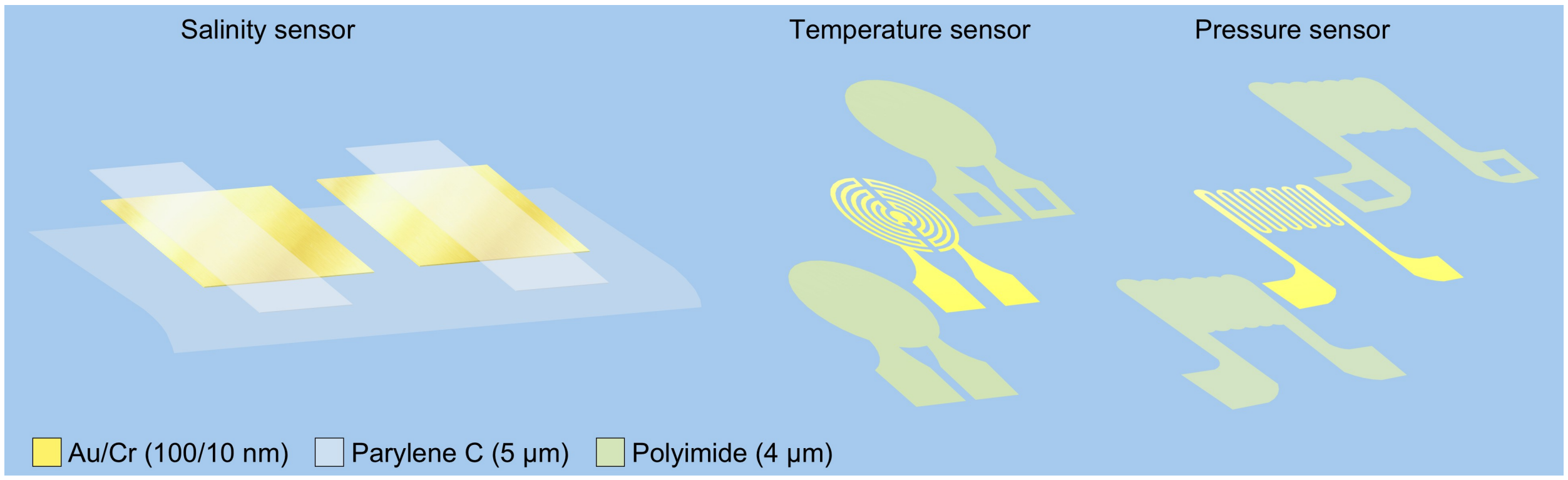

Fig. S1. Exploded views showing the multilayer structures of the salinity, temperature and pressure sensors.

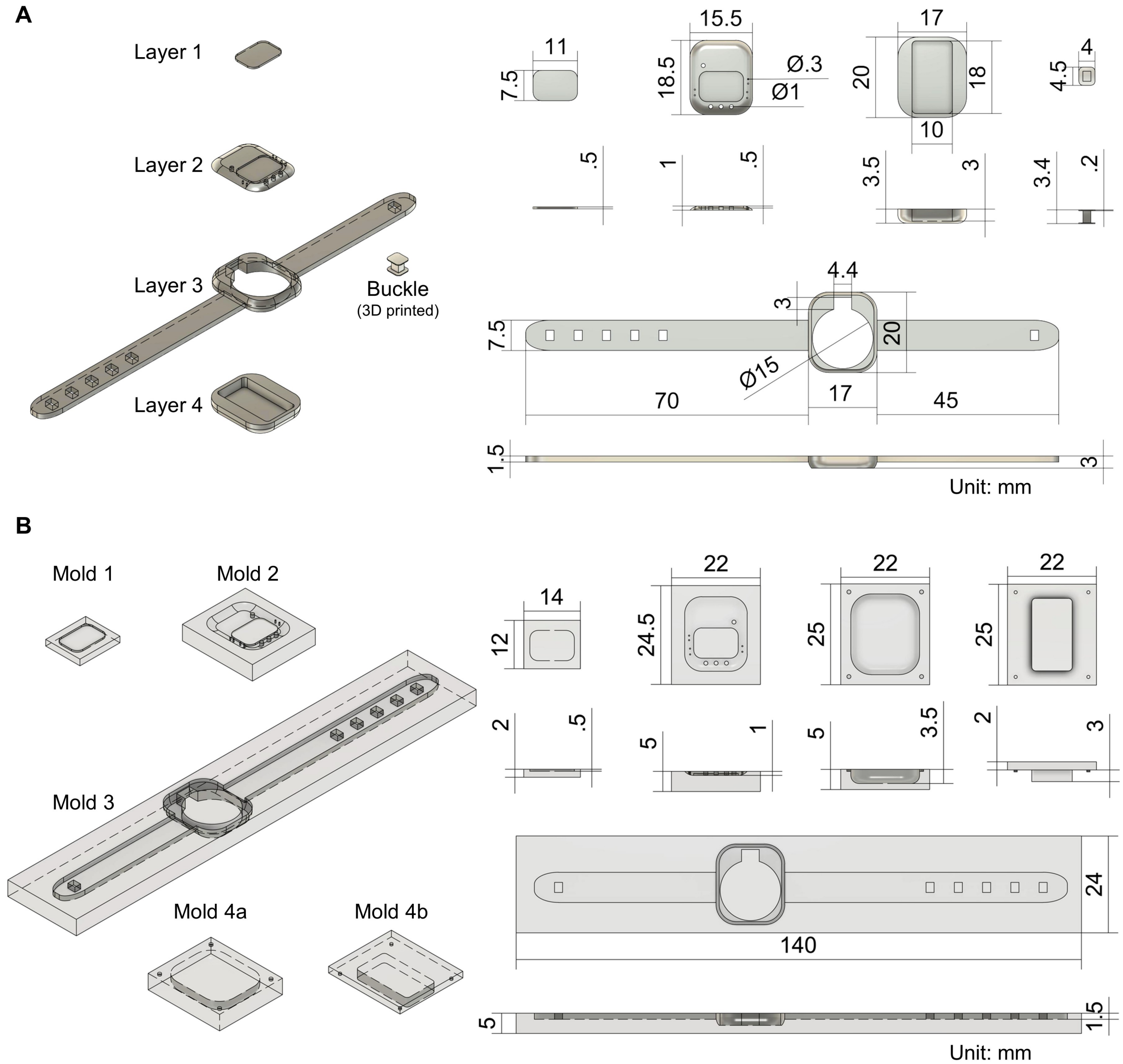

Fig. S2. Design of the wearable soft elastomer casing. 3D models, 2D drawings, and dimensions of **(A)** soft elastomer casing and **(B)** 3D printed molds.

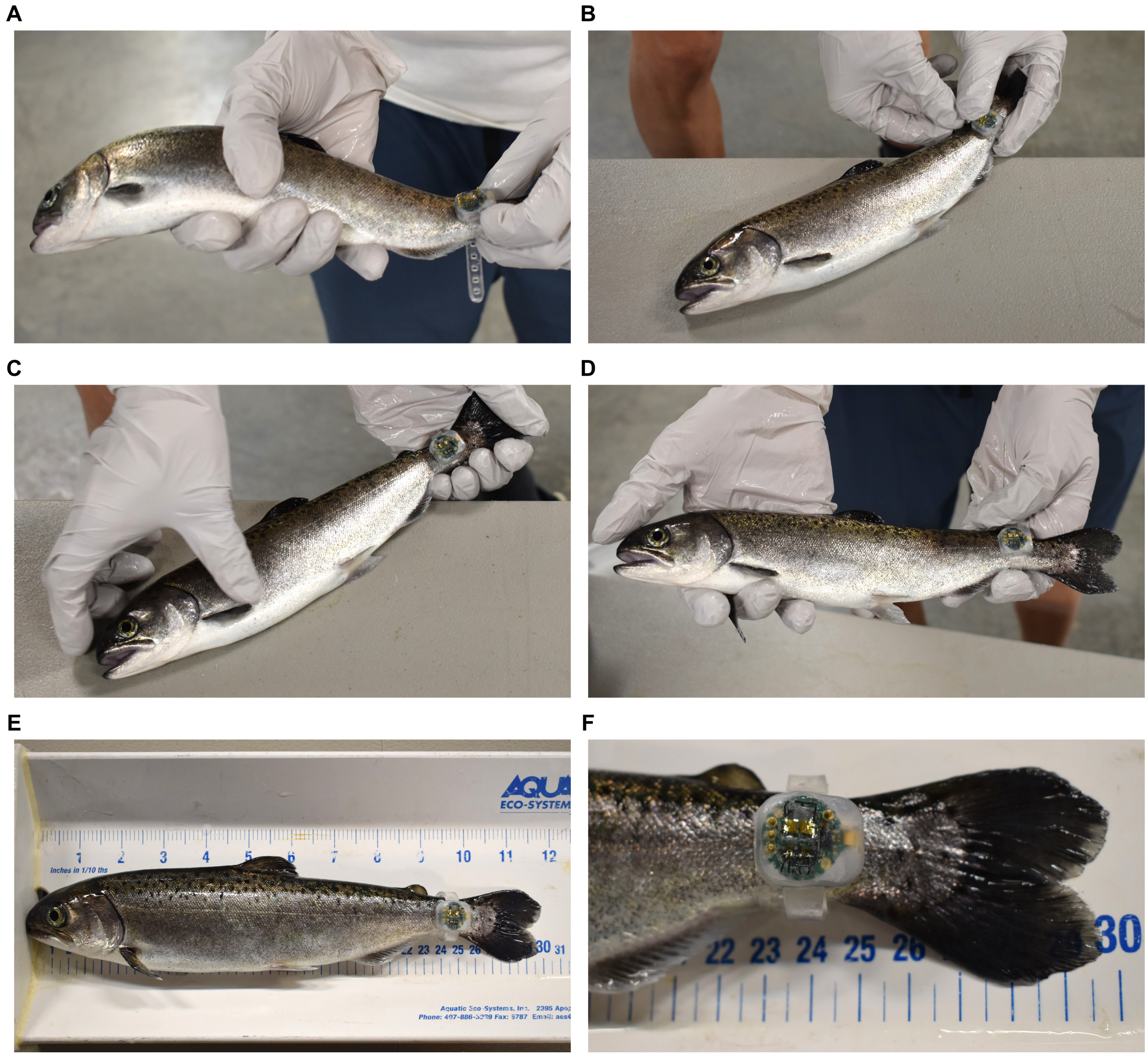

Fig. S3. Photos of a wearable ocean sensing tag attached to a juvenile Chinook salmon. (A) The wearable ocean sensing tag can be secured on the fish's tail with its watch-style band. **(B)** Wrap the watch strap around the fish's tail and secure it with the buckle through the adjustment hole. **(C)** Completed tagging and **(D)** holding the fish in hands. **(E)** The juvenile Chinook salmon measures approximately 30 cm (11.8 inches). **(F)** Zoomed-in view on the fish's tail, showing the dimensions of the wearable ocean sensing tag (2 cm x 1.7 cm).

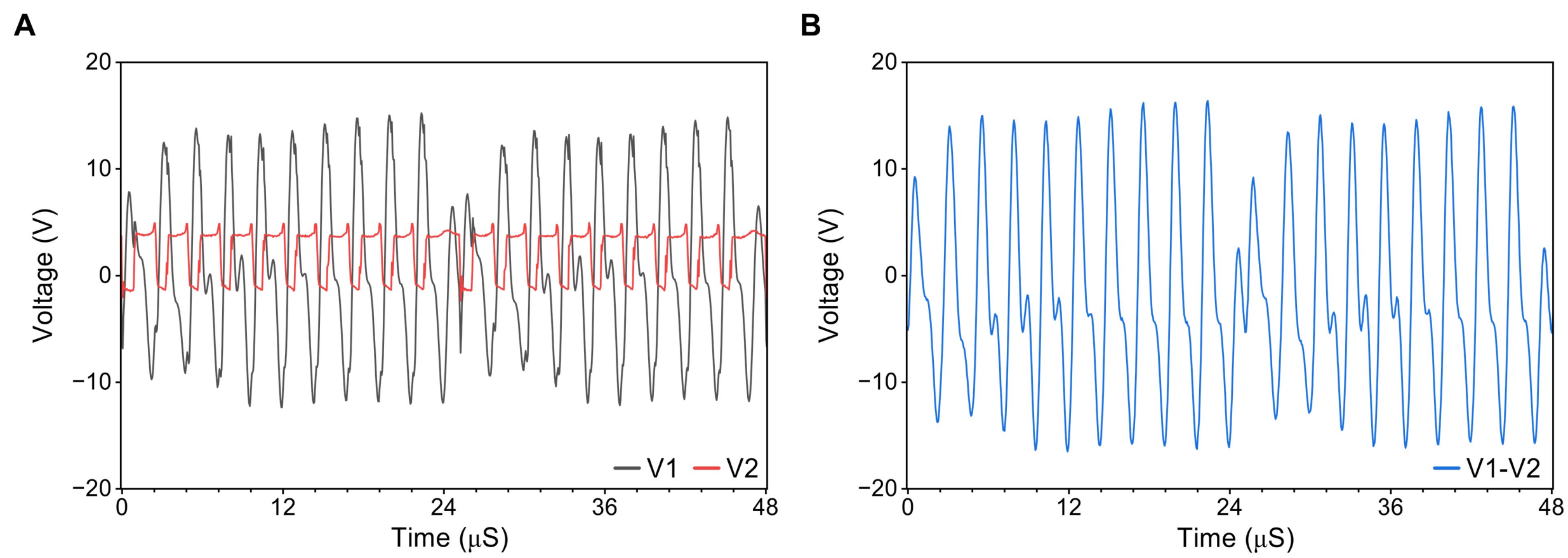

Fig. S4. Alternating voltages for driving the acoustic transducer. (A) Voltages applied to the inner and outer walls of the acoustic transducer, and **(B)** net voltage applied across the transducer.

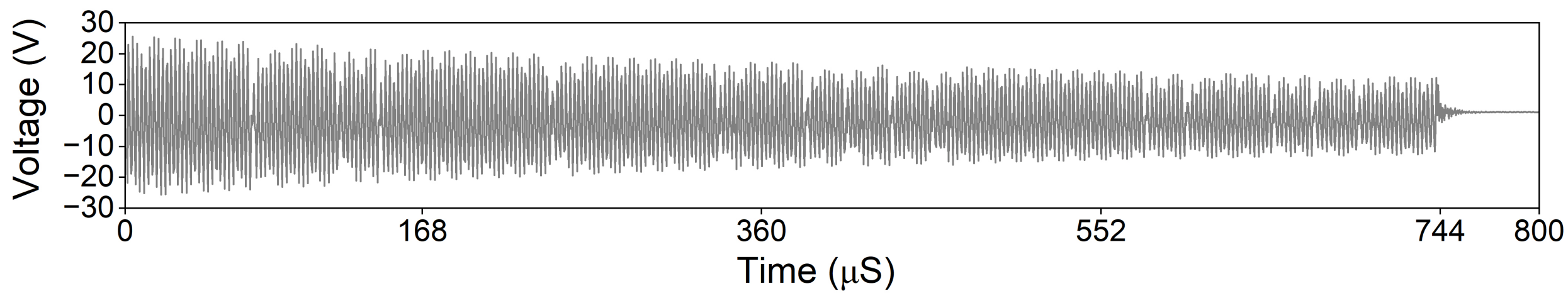

Fig. S5. Complete waveform from the acoustic transmission module, showing the 744 μs data transmission and residual signal.

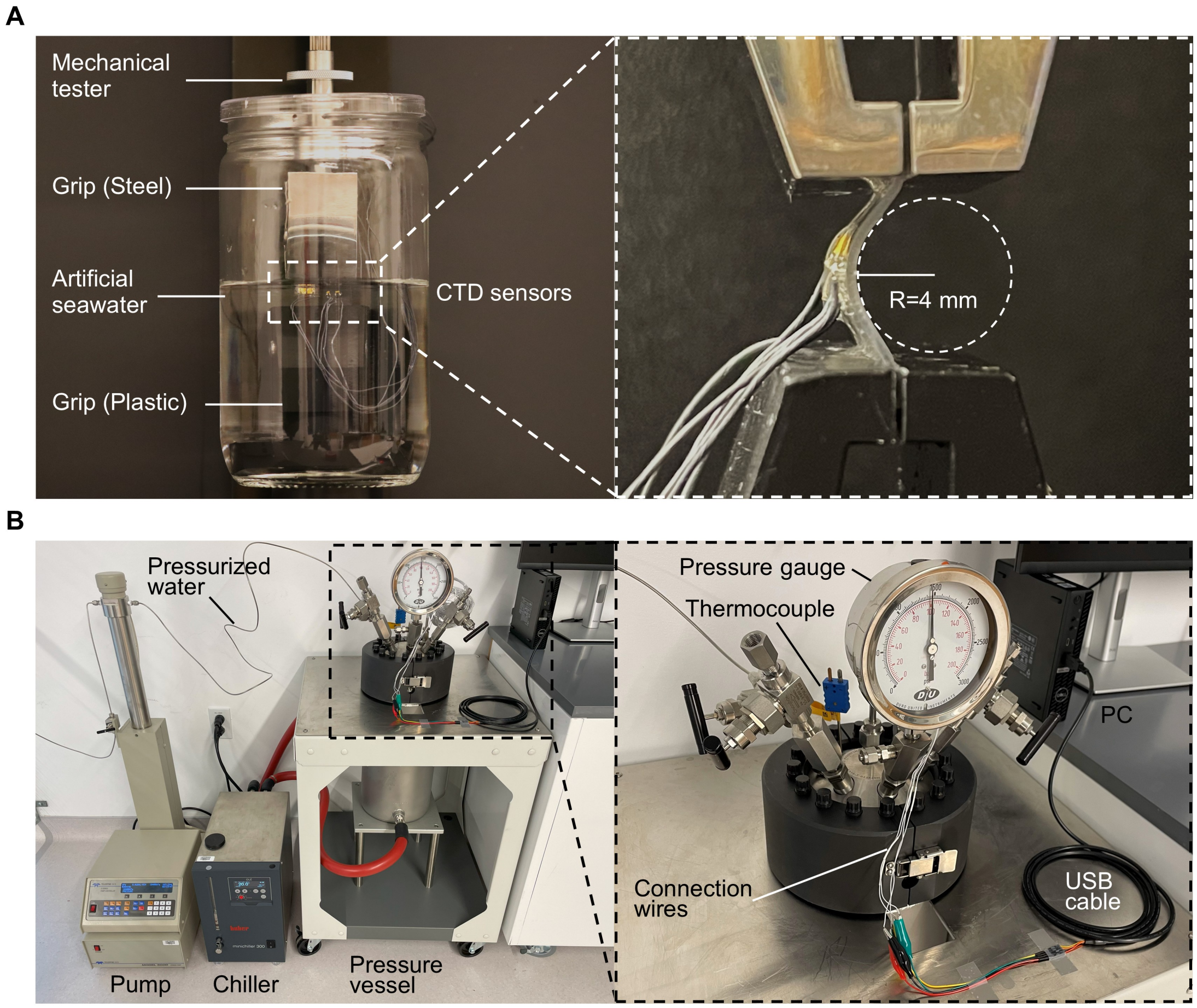

Fig. S6. Experimental setup for reliability tests. The reliability of the CTD sensors and ocean sensing tag were evaluating through **(A)** cyclic bending and **(B)** cyclic pressurization and pressure tolerance tests.

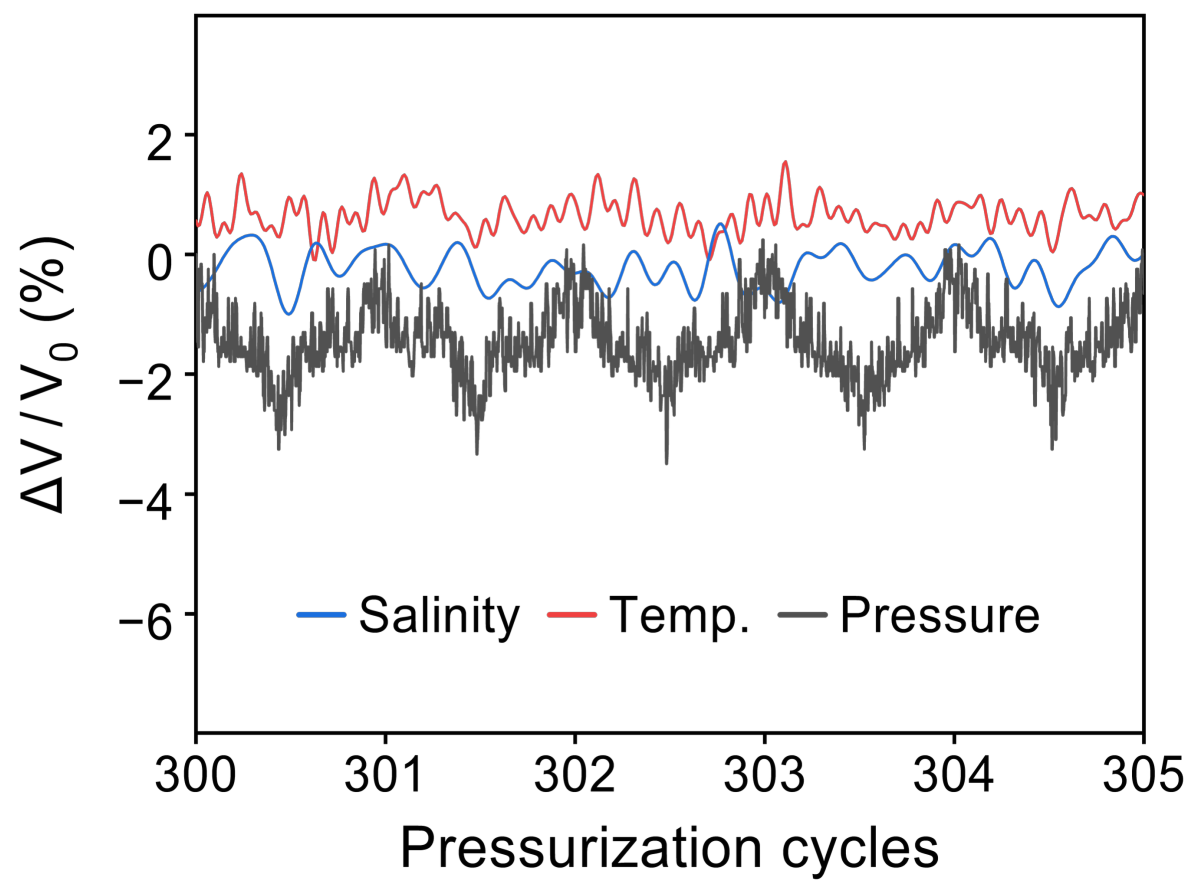

Fig. S7. Cyclic pressurization test results. Zoomed-in view of the relative voltage changes of CTD sensors during the 300th to 304th cyclic pressurization tests between 0.1 and 15 MPa.

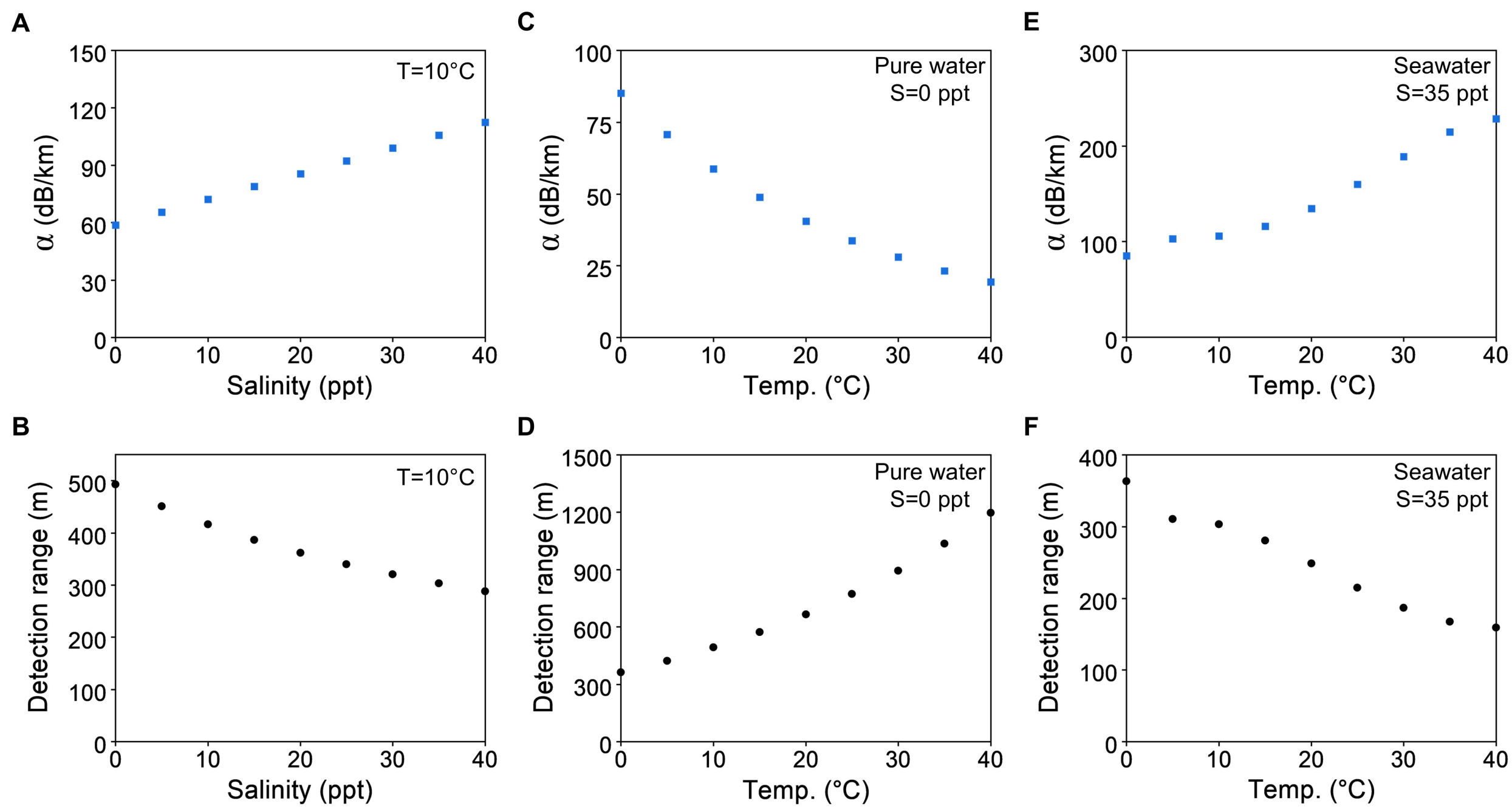

Fig. S8. Environmental effects on the absorption coefficient (α) and detection range of acoustic telemetry at the maximum source level of 164.3 dB. (A, B) Effect of salinity at a constant temperature of 10°C. **(C, D)** Effect of temperature in pure water. **(E, F)** Effect of temperature in seawater at a constant salinity of 35 ppt.

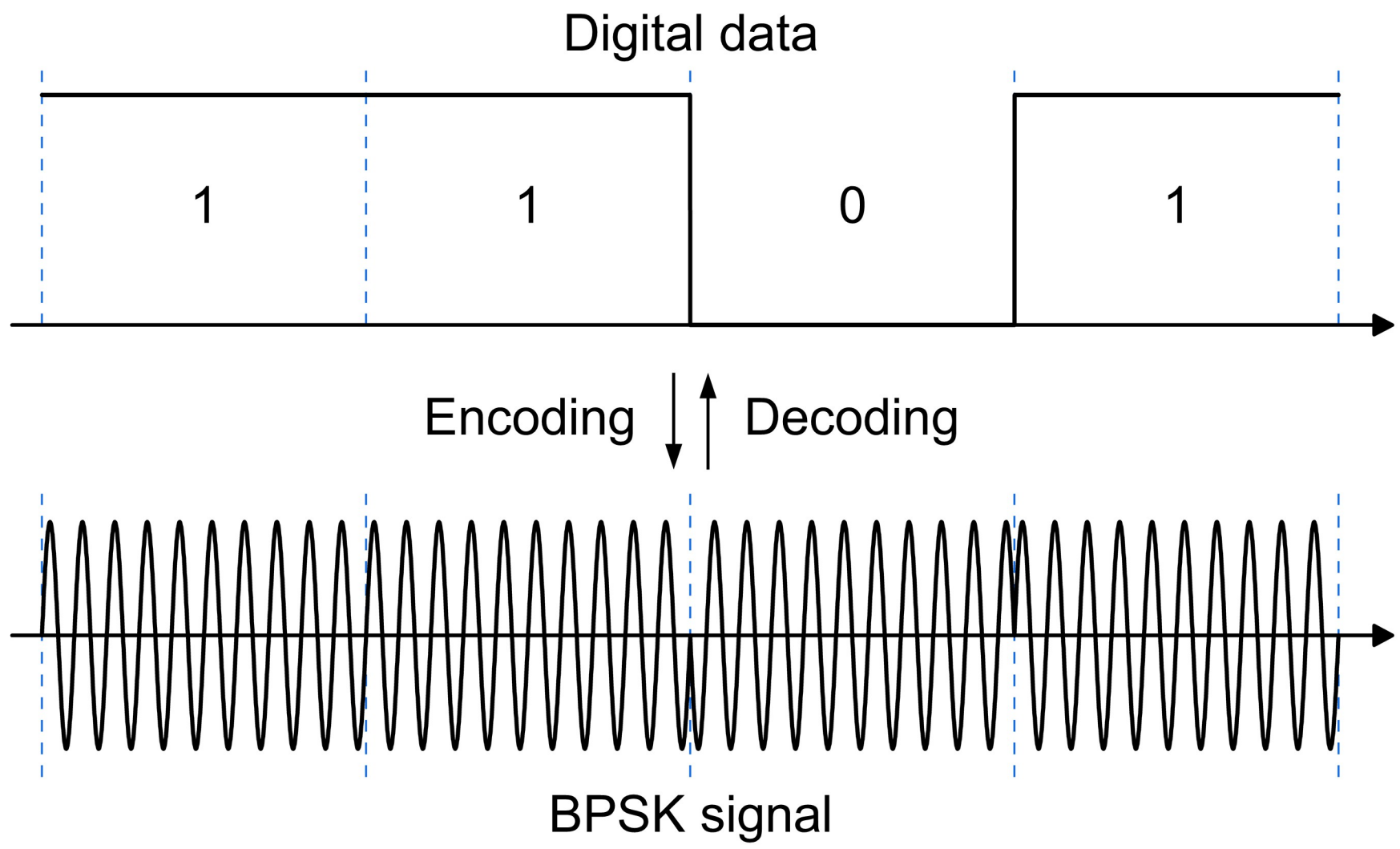

Fig. S9. Schematic of data modulation between digital and BPSK signals. In BPSK modulation, no phase change in the carrier ultrasound represents a 1-to-1 or 0-to-0 transition, while a phase change indicates a 1-to-0 or 0-to-1 transition.

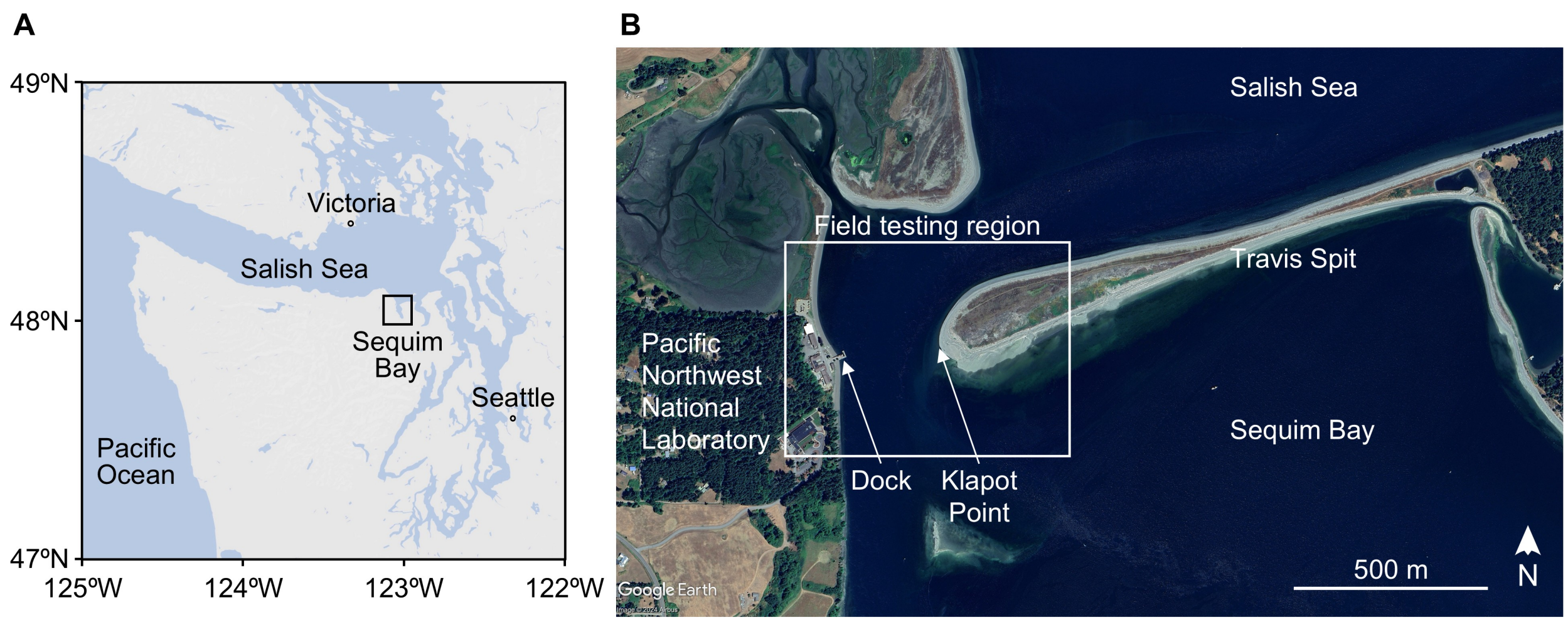

Fig. S10. Field-testing region. Satellite maps showing **(A)** the location of the field-testing region and **(B)** its relative geographic position.

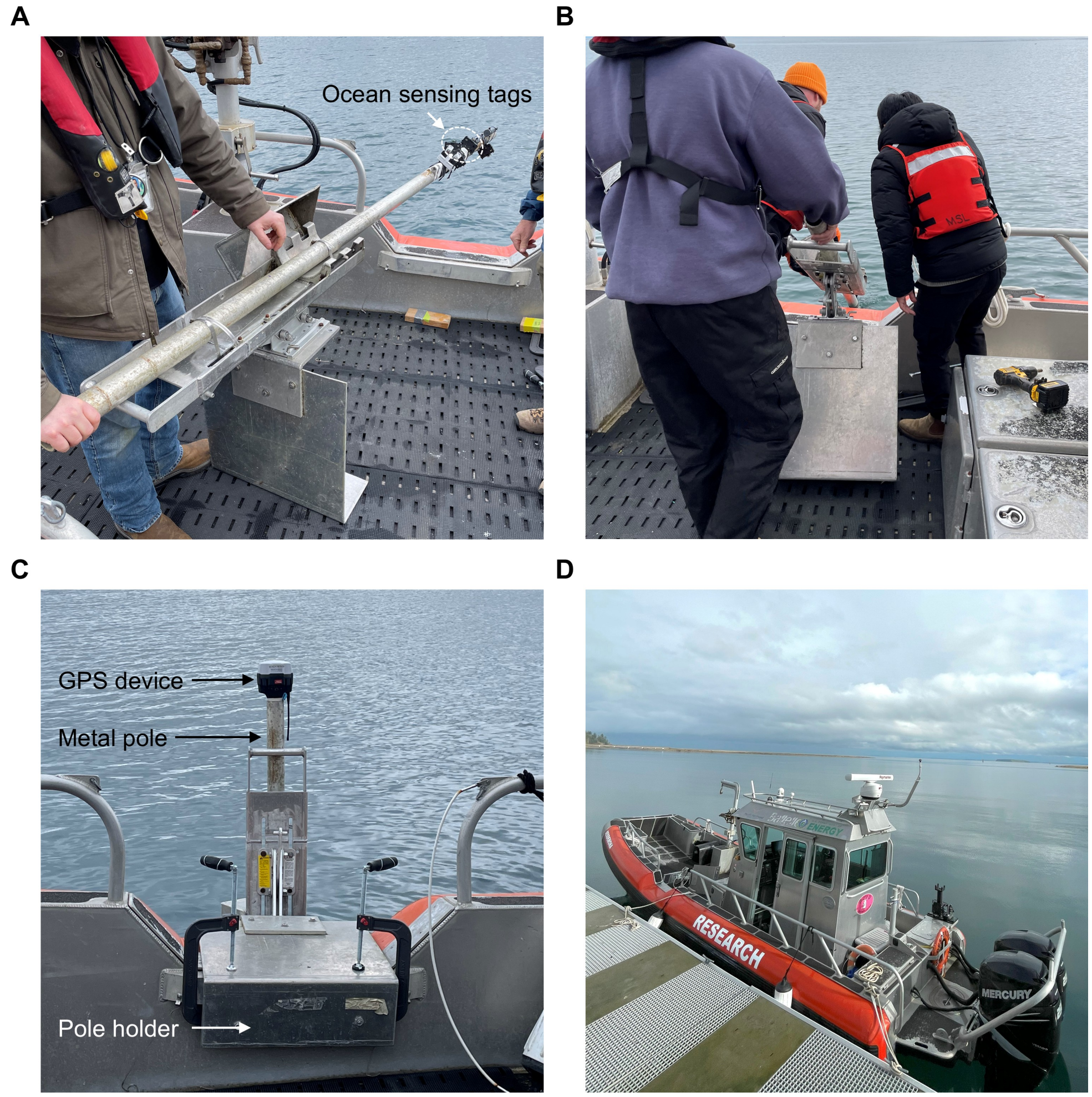

Fig. S11. Preparation for field testing. (A) Attaching devices to the metal pole and installing the pole into the pole holder. **(B)** Immersing the pole in the ocean and securing the holder to the boat. **(C)** Placing a GPS tracker on top of the metal pole. **(D)** Leaving the floating dock and starting the testing.

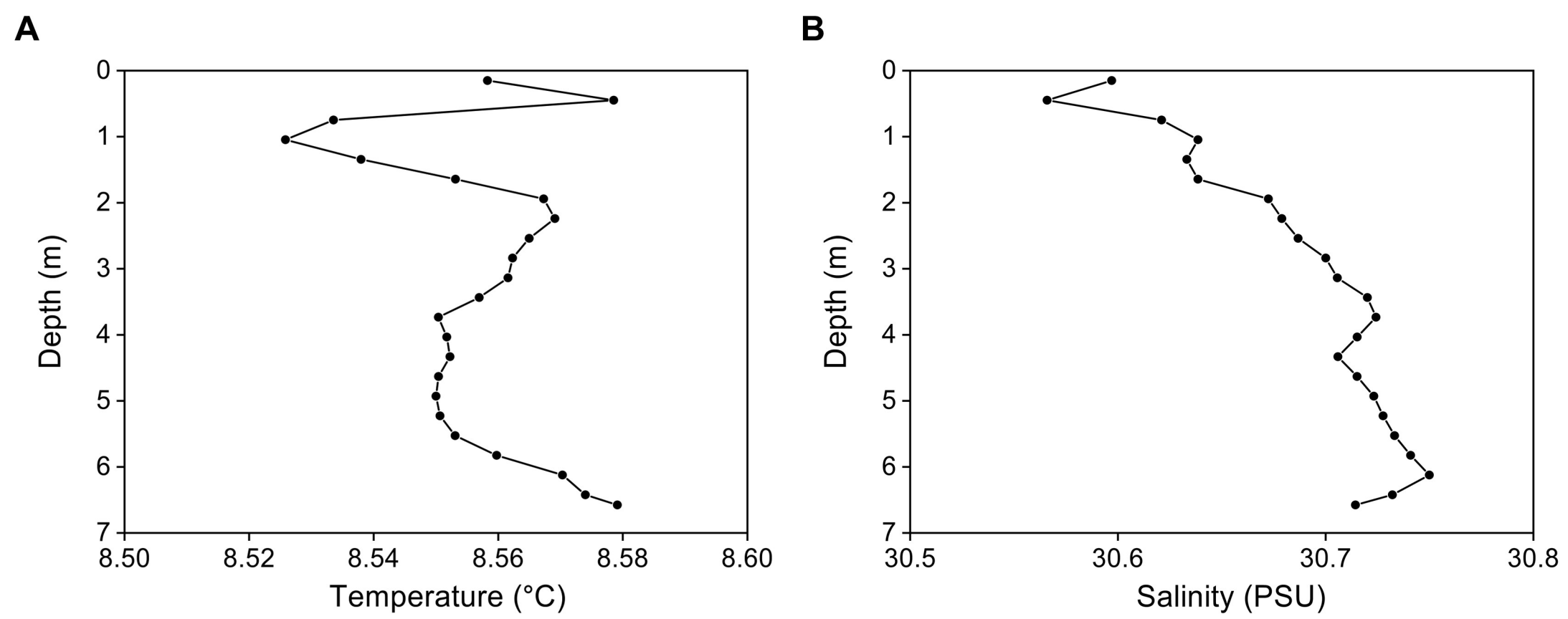

Fig. S12. Vertical CTD profiles. (A) Temperature and **(B)** salinity as a function of depth, recorded by a commercial sensor (SonTek CastAway-CTD) on the first day of field testing (Feb. 21, 2024).

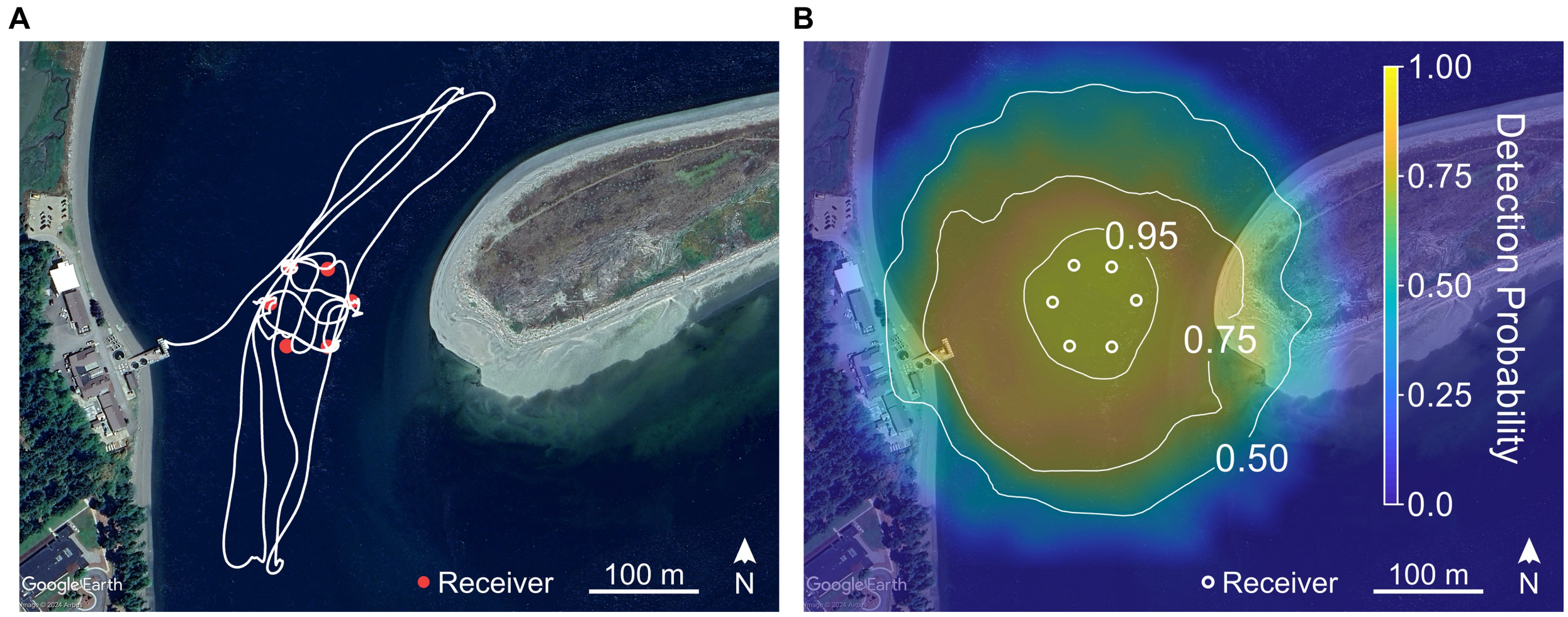

Fig. S13. Detection probability for acoustic transmission. Satellite maps showing **(A)** the boat route on the second day of field testing (Feb. 22, 2024), and **(B)** the contour map of detection probability for acoustic transmission.

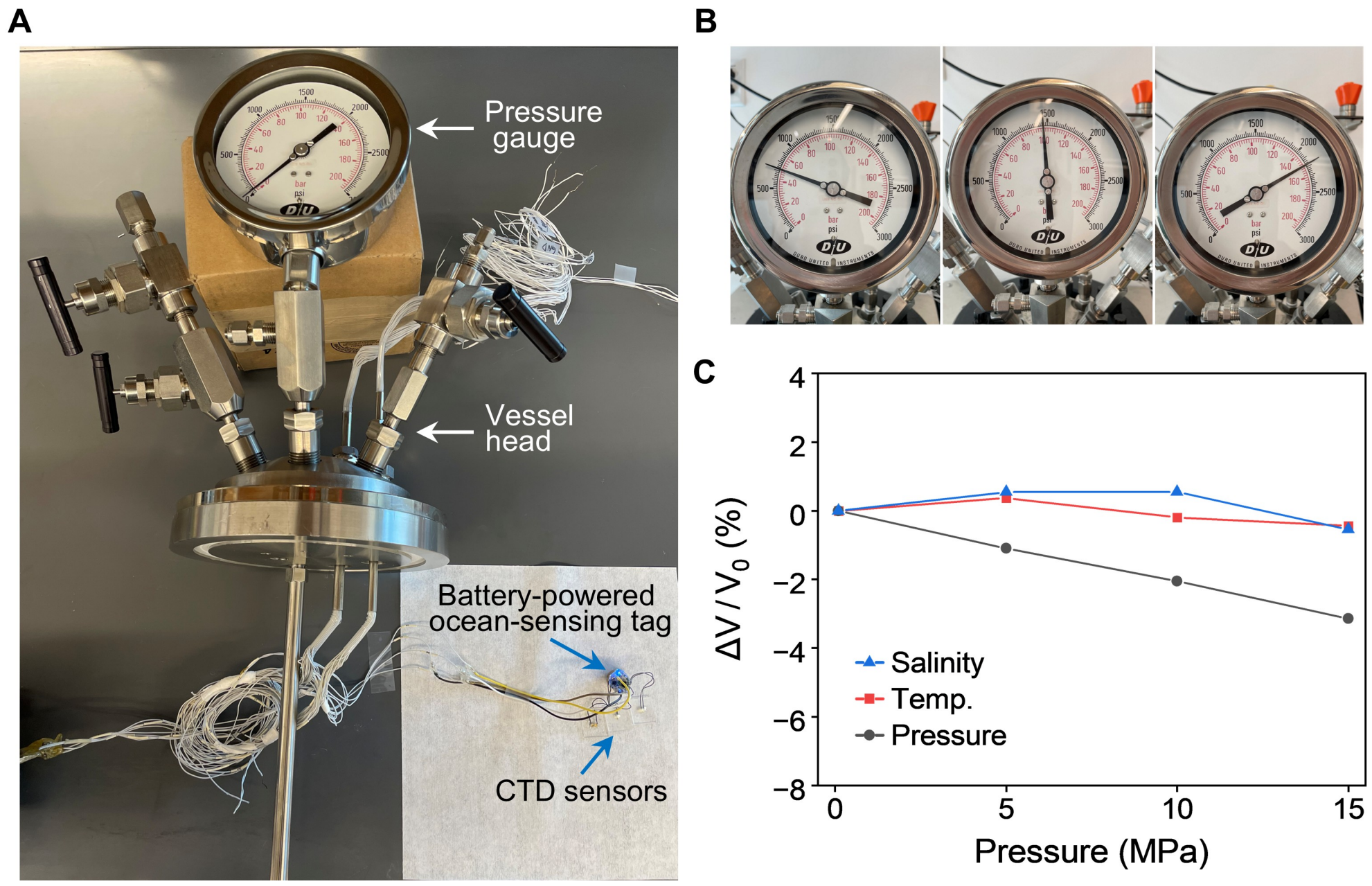

Fig. S14. Pressure tolerance testing of the battery-powered ocean-sensing tag. (A) Experimental setup for testing a battery-powered ocean-sensing tag in a custom-built pressure vessel system. **(B)** Photos of the pressure gauge during pressurization, showing the controlled hydrostatic pressures of 50, 100 and 150 bar (corresponding to 5.0, 10.0, and 15.0 MPa). **(C)** Pressurization test results of the complete battery-powered device.

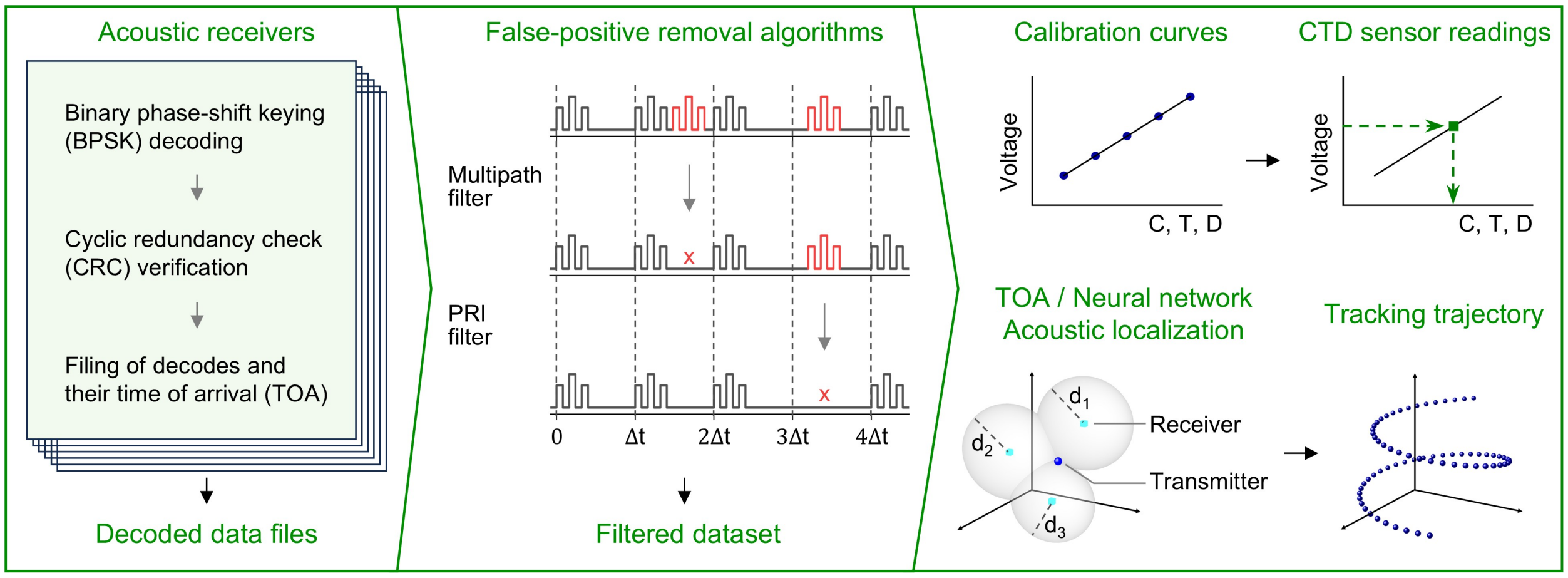

Fig. S15. Schematic of data processing steps for computing sensor readings and tracking trajectory. The process includes acoustic data decoding by autonomous acoustic receivers, false-positive removal by filtering algorithms, sensor calibration, and acoustic localization based on time-of-arrival (TOA) data.

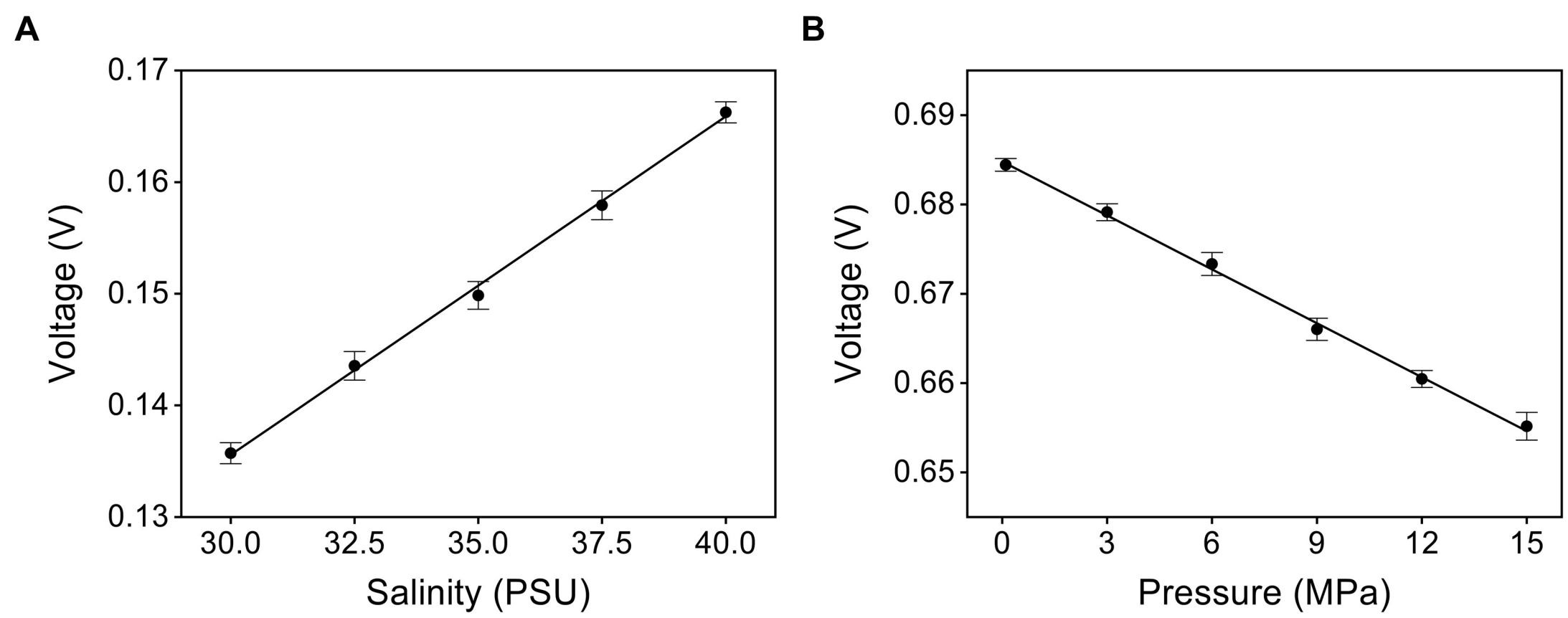

Fig. S16. Sensor performance at low temperature. Calibration curves of **(A)** salinity and **(B)** pressure sensors at 8.5°C.

Table S1. Comparison of existing CTD devices with our ocean-sensing tag.

	**SBE 49 CTD** **(*69, 70*)**	**CTD-SRDL** **(*1, 71, 72*)**	**DST CTD** **(*49*)**	**CTD biotag** **(*50, 73, 74*)**	**Marine skin** **(*24, 25*)**	**Ocean sensing tag** **(this work)**
Device images	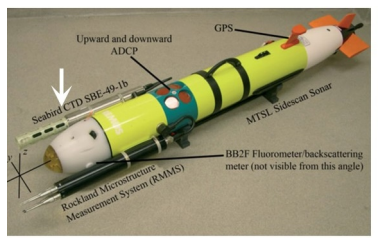	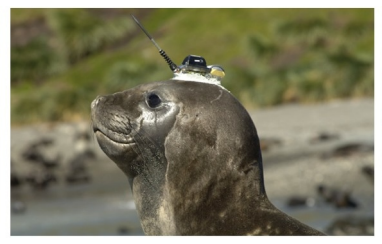	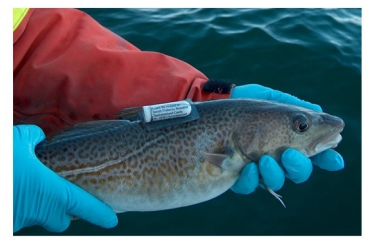	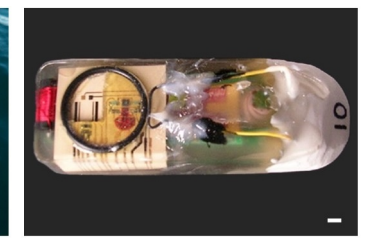	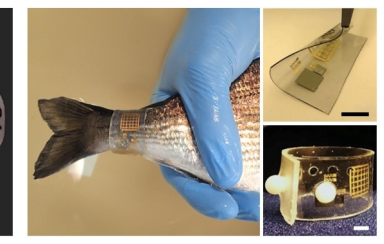	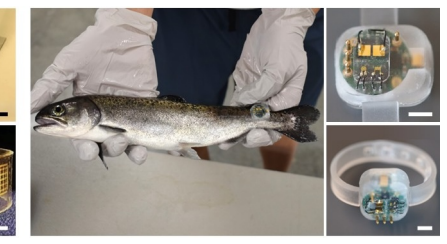
Size (LxWxH)	62 mm x 620 mm (⌀ x L)	105 mm x 70 mm x 40 mm	15 mm x 50 mm (⌀ x L)	100 mm x 43 mm x 24 mm	20 mm x 20 mm x 0.3 mm (w/o bracelet)	20 mm x 17 mm x 7.5 mm
Need for housing	Yes (Titanium)	Yes (Epoxy)	Yes (Alumina)	Yes (Urethane)	No	No
Weight (in air)	2.7 kg	545 g	21 g	104 g	3 g	4.9 g
Real-time data transmission	No (Data logged to AUV, retrieved after recovery)	No (Memory storage, Satellite relay upon resurfacing)	No (Memory storage, RF transfer after recovery)	No (Memory storage, Wi-Fi transfer after recovery)	No (Memory storage, Bluetooth transfer after recovery)	Yes (Underwater acoustic telemetry and memory storage)
Field evaluation	Monterey Bay (Pacific Ocean)	Southern Ocean & Arctic Ocean	Northeast Atlantic	Gulf of Mexico	-	Sequim Bay, Salish Sea (Pacific Ocean)
Application scope	AUV-mounted, no animal use	Compatible with seals and turtles	Fish-deployable	Compatible with turtles	Fish-deployable	Various platforms: animal tags, divers, and soft robotics
Attachment method	-	Epoxy glued	Wire-threaded (invasive)	Epoxy glued	Wearable bracelet (noninvasive)	Wearable watch (noninvasive)
			Conductivity (salinity) measurement			
Range:	0 to 9 S/m	0 to 80 mS/cm	3 to 68 mS/cm [a]	8.96 to 40.32 PSU	5 to 45 PSU	30 to 40 PSU
Accuracy:	± 0.0003 S/m	± 0.01 mS/cm	±1 PSU [a]	±0.21 / 0.14 PSU [b]	-	±1.140 PSU [c]
Resolution:	0.00005 S/m	0.002 mS/cm	0.02 PSU [a]	-	-	0.233 PSU [d]
			Temperature measurement			
Range:	-5 to 35°C	-5 to 35°C	-1 to 40°C	0 to 35°C	5 to 60°C	5 to 40°C
Accuracy:	± 0.002°C	± 0.005°C	± 0.1°C	± 0.35 / 0.33°C [b]	-	± 0.233°C [c]
Resolution:	0.0001°C	0.001°C	0.032°C	-	-	0.0488°C [d]
			Depth (pressure) measurement			
Range:	0-7,000/10,500 m	2 to 2,000 dbar	10 to 2,400 m	0 to 110 dbar	500 to 1,500 m	0 to 1,500 m
Accuracy:	± 0.1% of range	± 2 dbar	± 0.6% of range	± 2.0%	-	-
Resolution:	0.002% of range	0.05 dbar	0.03% of range	-	-	2.5% of range [d]
Cost	$18,500 [e]	$7,500 [e]	$1,375 [e]	Research prototype	Research prototype	Estimated: $300-$600

Scale bars: 5 mm. Figures reproduced with permission from (*69*) (copyright 2009 Inter-Research), (*71*) (copyright 2018 Elsevier), (*49*) (copyright 2010 Inter-Research), (*50*) (copyright 2013 Elsevier), and (*24*) (copyright 2019 Wiley). The original figures were not published using a Creative Commons type license.

[a] Data are based on the specifications of the wide-range model.

[b] Accuracy is calculated as the mean of the absolute differences between the biotag and two commercial instruments (Neil Brown ATB 1250 and RBR XR-420 CTD) (*50*).

[c] Accuracy is calculated as the mean of the absolute differences between the tag's sensor data and reference values from a commercial SonTek CastAway-CTD, both recorded during field testing in the Salish Sea.

[d] Resolution is calculated as the physical change per step of a 12-bit dataset, representing the smallest change the tag can detect.

[e] Prices are listed in USD for reference only and may vary depending on the manufacturer, supplier, and purchasing conditions.

Table S2. Comparison of CTD measurements recorded with our ocean-sensing tag and a commercial CTD sensor during field testing.

	Salinity (PSU)	Temperature (°C)	Pressure (MPa)	Overall
Our ocean-sensing tag (Mean values)	31.369	8.464	0.0115	-
Reference (SonTek CastAway-CTD)	30.636	8.532	0.0120	-
Relative error	2.39%	-0.79%	-4.16%	-
Detection efficiency (Acoustic telemetry)	93.53%	86.67%	81.45%	87.34%

Table S3. Tracking efficiency and accuracy of underwater acoustic localization.

	Efficiency	Mean (X)	Mean (Y)	Median (X)	Median (Y)	RMS (X)	RMS (Y)
Track 1	80.24%	1.40	1.46	1.08	1.06	1.84	1.86
Track 2	80.16%	0.58	1.10	0.55	1.03	0.67	1.35
Track 3	80.11%	0.81	1.22	0.81	1.07	0.93	1.48
Overall	80.20%	1.14	1.35	0.90	1.05	1.54	1.71

* Accuracy is evaluated based on the mean absolute error, median absolute error, and root mean square (RMS) error of the differences between acoustic localization and GPS measurements.

REFERENCES

1. R. Harcourt, A. M. M. Sequeira, X. Zhang, F. Roquet, K. Komatsu, M. Heupel, C. McMahon, F. Whoriskey, M. Meekan, G. Carroll, S. Brodie, C. Simpfendorfer, M. Hindell, I. Jonsen, D. P. Costa, B. Block, M. Muelbert, B. Woodward, M. Weise, K. Aarestrup, M. Biuw, L. Boehme, S. J. Bograd, D. Cazau, J.-B. Charrassin, S. J. Cooke, P. Cowley, P. J. N. de Bruyn, T. Jeanniard du Dot, C. Duarte, V. M. Eguíluz, L. C. Ferreira, J. Fernández-Gracia, K. Goetz, Y. Goto, C. Guinet, M. Hammill, G. C. Hays, E. L. Hazen, L. A. Hückstädt, C. Huveneers, S. Iverson, S. A. Jaaman, K. Kittiwattanawong, K. M. Kovacs, C. Lydersen, T. Moltmann, M. Naruoka, L. Phillips, B. Picard, N. Queiroz, G. Reverdin, K. Sato, D. W. Sims, E. B. Thorstad, M. Thums, A. M. Treasure, A. W. Trites, G. D. Williams, Y. Yonehara, M. A. Fedak, Animal-borne telemetry: An integral component of the ocean observing toolkit. *Front. Mar. Sci.* **6**, 326 (2019).

2. M. L. Grundlingh, P. D. Morant, R. C. van Ballegooyen, A. Badenhorst, E. Gomes, L. Greyling, J. Guddal, I. T. Hunter, D. W. Japp, L. Maartens, K. R. Peard, G. G. Smith, C. K. Wainman, "Environmental data requirements of maritime operations in the Benguela coastal ocean," in *Large Marine Ecosystems,* V. Shannon, G. Hempel, P. Malanotte-Rizzoli, C. Moloney, J. Woods, Eds. (Elsevier, 2006), vol. 14, pp. 349–372.

3. E. Chassignet, H. Hurlburt, E. J. Metzger, O. Smedstad, J. Cummings, G. Halliwell, R. Bleck, R. Baraille, A. Wallcraft, C. Lozano, H. Tolman, A. Srinivasan, S. Hankin, P. Cornillon, R. Weisberg, A. Barth, R. He, F. Werner, J. Wilkin, US GODAE: Global ocean prediction with the hybrid coordinate ocean model (HYCOM). *Oceanography* **22**, 64–75 (2009).

4. W. Kang, T. Mittal, S. Bire, J. M. Campin, J. Marshall, How does salinity shape ocean circulation and ice geometry on Enceladus and other icy satellites? *Sci. Adv.* **8**, eabm4665 (2022).

5. J. Raddatz, V. Liebetrau, A. Ruggeberg, A. Foubert, S. Flogel, D. Nurnberg, K. Hissmann, J. Musiol, T. J. Goepfert, A. Eisenhauer, W. C. Dullo, Living on the edge: Environmental variability of a shallow late Holocene cold-water coral mound. *Coral Reefs* **41**, 1255–1271 (2022).

6. E. W. Doddridge, J. Marshall, H. Song, J. M. Campin, M. Kelley, L. Nazarenko, Eddy compensation dampens Southern Ocean sea surface temperature response to westerly wind trends. *Geophys. Res. Lett.* **46**, 4365–4377 (2019).

7. A. P. S. Wong, S. E. Wijffels, S. C. Riser, S. Pouliquen, S. Hosoda, D. Roemmich, J. Gilson, G. C. Johnson, K. Martini, D. J. Murphy, M. Scanderbeg, T. V. S. U. Bhaskar, J. J. H. Buck, F. Merceur, T. Carval, G. Maze, C. Cabanes, X. André, N. Poffa, I. Yashayaev, P. M. Barker, S. Guinehut, M. Belbéoch, M. Ignaszewski, M. O. N. Baringer, C. Schmid, J. M. Lyman, K. E. McTaggart, S. G. Purkey, N. Zilberman, M. B. Alkire, D. Swift, W. B. Owens, S. R. Jayne, C. Hersh, P. Robbins, D. West-Mack, F. Bahr, S. Yoshida, P. J. H. Sutton, R. Cancouët, C. Coatanoan, D. Dobbler, A. G. Juan, J. Gourrion, N. Kolodziejczyk, V. Bernard, B. Bourlès, H. Claustre, F. D'Ortenzio, S. Le Reste, P.-Y. Le Traon, J.-P. Rannou, C. Saout-Grit, S. Speich, V. Thierry, N. Verbrugge, I. M. Angel-Benavides, B. Klein, G. Notarstefano, P.-M. Poulain, P. Vélez-Belchí, T. Suga, K. Ando, N. Iwasaska, T. Kobayashi, S. Masuda, E. Oka, K. Sato, T. Nakamura, K. Sato, Y. Takatsuki, T. Yoshida, R. Cowley, J. L. Lovell, P. R. Oke, E. M. van Wijk, F. Carse, M. Donnelly, W. J. Gould, K. Gowers, B. A. King, S. G. Loch, M. Mowat, J. Turton, E. P. Rama Rao, M. Ravichandran, H. J. Freeland, I. Gaboury, D. Gilbert, B. J. W. Greenan, M. Ouellet, T. Ross, A. Tran, M. Dong, Z. Liu, J. Xu, K. Kang, H. Jo, S.-D. Kim, H.-M. Park, Argo data 1999–2019: Two million temperature-salinity profiles and subsurface velocity observations from a global array of profiling floats. *Front. Mar. Sci.* **7**, 700 (2020).

8. A. G. O'Carroll, E. M. Armstrong, H. M. Beggs, M. Bouali, K. S. Casey, G. K. Corlett, P. Dash, C. J. Donlon, C. L. Gentemann, J. L. Høyer, A. Ignatov, K. Kabobah, M. Kachi, Y. Kurihara, I. Karagali, E. Maturi, C. J. Merchant, S. Marullo, P. J. Minnett, M. Pennybacker, B. Ramakrishnan, R. Ramsankaran, R. Santoleri, S. Sunder, S. Saux Picart, J. Vázquez-Cuervo, W. Wimmer, Observational needs of sea surface temperature. *Front. Mar. Sci.* **6**, 420 (2019).

9. D. Roemmich, J. T. Sherman, R. E. Davis, K. Grindley, M. McClune, C. J. Parker, D. N. Black, N. Zilberman, S. G. Purkey, P. J. H. Sutton, J. Gilson, Deep SOLO: A full-depth profiling float for the Argo program. *J. Atmos. Oceanic Tech.* **36**, 1967–1981 (2019).

10. A. N. Grekov, N. A. Grekov, E. N. Sychov, Measuring salinity and density of seawater samples with different salt compositions and suspended materials. *Metrology* **1**, 107–121 (2021).

11. K. S. Johnson, H. W. Jannasch, L. J. Coletti, V. A. Elrod, T. R. Martz, Y. Takeshita, R. J. Carlson, J. G. Connery, Deep-sea DuraFET: A pressure tolerant pH sensor designed for global sensor networks. *Anal. Chem.* **88**, 3249–3256 (2016).

12. S. McPhail, Autosub6000: A deep diving long range AUV. *J. Bionic Eng.* **6**, 55–62 (2009).

13. R. B. Wynn, V. A. I. Huvenne, T. P. Le Bas, B. J. Murton, D. P. Connelly, B. J. Bett, H. A. Ruhl, K. J. Morris, J. Peakall, D. R. Parsons, E. J. Sumner, S. E. Darby, R. M. Dorrell, J. E. Hunt, Autonomous underwater vehicles (AUVs): Their past, present and future contributions to the advancement of marine geoscience. *Mar. Geol.* **352**, 451–468 (2014).

14. Z. Duguid, R. Camilli, Improving resource management for unattended observation of the marginal ice zone using autonomous underwater gliders. *Front. Robot. AI* **7**, 579256 (2021).

15. K. Taira, D. Yanagimoto, S. Kitagawa, Deep CTD casts in the challenger deep, Mariana Trench. *J. Oceanogr.* **61**, 447–454 (2005).

16. S. Xiao, M. Zhang, C. Liu, C. Jiang, X. Wang, F. Yang, CTD sensors for ocean investigation including state of art and commercially available. *Sensors* **23**, 586 (2023).

17. L. Boehme, P. Lovell, M. Biuw, F. Roquet, J. Nicholson, S. E. Thorpe, M. P. Meredith, M. A. Fedak, Technical note: Animal-borne CTD-satellite relay data loggers for real-time oceanographic data collection. *Ocean Sci.* **5**, 685–695 (2009).

18. T. Photopoulou, M. A. Fedak, J. Matthiopoulos, B. McConnell, P. Lovell, The generalized data management and collection protocol for conductivity-temperature-depth satellite relay data loggers. *Anim. Biotelemetry* **3**, 21 (2015).

19. C. R. McMahon, F. Roquet, S. Baudel, M. Belbeoch, S. Bestley, C. Blight, L. Boehme, F. Carse, D. P. Costa, M. A. Fedak, C. Guinet, R. Harcourt, E. Heslop, M. A. Hindell, X. Hoenner, K. Holland, M. Holland, F. R. A. Jaine, T. Jeanniard du Dot, I. Jonsen, T. R. Keates, K. M. Kovacs, S. Labrousse, P. Lovell, C. Lydersen, D. March, M. Mazloff, M. K. McKinzie, M. M. C. Muelbert, K. O'Brien, L. Phillips, E. Portela, J. Pye, S. Rintoul, K. Sato, A. M. M. Sequeira, S. E. Simmons, V. M. Tsontos, V. Turpin, E. van Wijk, D. Vo, M. Wege, F. G.

Whoriskey, K. Wilson, B. Woodward, Animal borne ocean sensors–AniBOS–An essential component of the global ocean observing system. *Front. Mar. Sci.* **8**, 751840 (2021).

20. D. E. Fullenkamp, W. Y. Maeng, S. Oh, H. Luan, K. S. Kim, I. A. Chychula, J. T. Kim, J. Y. Yoo, C. W. Holgren, A. R. Demonbreun, S. George, B. Li, Y. Hsu, G. Chung, J. Yoo, J. Koo, Y. Park, I. R. Efimov, E. M. McNally, J. A. Rogers, Simultaneous electromechanical monitoring in engineered heart tissues using a mesoscale framework. *Sci. Adv.* **10**, eado7089 (2024).

21. F. Yi, X. Wang, S. Niu, S. Li, Y. Yin, K. Dai, G. Zhang, L. Lin, Z. Wen, H. Guo, J. Wang, M. H. Yeh, Y. Zi, Q. Liao, Z. You, Y. Zhang, Z. L. Wang, A highly shape-adaptive, stretchable design based on conductive liquid for energy harvesting and self-powered biomechanical monitoring. *Sci. Adv.* **2**, e1501624 (2016).

22. N. T. Quirion, M. Madrid, J. Chang, A. Fehr, E. Rytkin, N. Shields, B. Burke, A. Elekeokwuri, I. R. Efimov, L. Lu, A soft multimodal optoelectronic array interface for multiparametric mapping of heart function in vivo. *Sci. Adv.* **11**, eads8608 (2025).

23. M. Filius, L. Fasching, R. van Wee, A. Y. Rwei, C. Joo, Decoding aptamer-protein binding kinetics for continuous biosensing using single-molecule techniques. *Sci. Adv.* **11**, eads9687 (2025).

24. S. F. Shaikh, H. F. Mazo-Mantilla, N. Qaiser, S. M. Khan, J. M. Nassar, N. R. Geraldi, C. M. Duarte, M. M. Hussain, Noninvasive featherlight wearable compliant “marine skin”: Standalone multisensory system for deep-sea environmental monitoring. *Small* **15**, e1804385 (2019).

25. J. M. Nassar, S. M. Khan, S. J. Velling, A. Diaz-Gaxiola, S. F. Shaikh, N. R. Geraldi, G. A. Torres Sevilla, C. M. Duarte, M. M. Hussain, Compliant lightweight non-invasive standalone “marine skin” tagging system. *npj Flex. Electron.* **2**, 13 (2018).

26. R. J. Lennox, E. M. Hanssen, E. S. Normann, B. T. Barlaup, C. I. Nilsen, L. S. Dahlmo, S. Berhe, K. W. Vollset, Migratory contingents of brown trout reveal variable exposure to anthropogenic threats along a fjord-river continuum. *Mar. Ecol.* **45**, e12820 (2024).

27. A. P. Klimley, R. Arauz, S. Bessudo, E. J. Chávez, N. Chinacalle, E. Espinoza, J. Green, A. R. Hearn, M. E. Hoyos-Padilla, E. Nalesso, J. T. Ketchum, C. Fischer, F. Ladino, G. Shillinger, G. Soler, T. Steiner, C. Peñaherrera-Palma, Studies of the movement ecology of sharks justify the existence and expansion of marine protected areas in the Eastern Pacific Ocean. *Environ. Biol. Fishes* **105**, 2133–2153 (2022).

28. B. Yang, J. Yu, T. Ma, A charge-free and membrane-free hybrid capacitive mixing system for salinity gradient energy harvesting. *J. Mater. Chem. A* **11**, 3388–3398 (2023).

29. G. Li, X. Chen, F. Zhou, Y. Liang, Y. Xiao, X. Cao, Z. Zhang, M. Zhang, B. Wu, S. Yin, Y. Xu, H. Fan, Z. Chen, W. Song, W. Yang, B. Pan, J. Hou, W. Zou, S. He, X. Yang, G. Mao, Z. Jia, H. Zhou, T. Li, S. Qu, Z. Xu, Z. Huang, Y. Luo, T. Xie, J. Gu, S. Zhu, W. Yang, Self-powered soft robot in the Mariana Trench. *Nature* **591**, 66–71 (2021).

30. R. K. Katzschmann, J. DelPreto, R. MacCurdy, D. Rus, Exploration of underwater life with an acoustically controlled soft robotic fish. *Sci. Robot.* **3**, eaar3449 (2018).

31. M.-A. Blanchet, C. Lydersen, R. A. Ims, K. M. Kovacs, Seasonal, oceanographic and atmospheric drivers of diving behaviour in a temperate seal species living in the high Arctic. *PLOS ONE* **10**, e0132686 (2015).

32. M. Horning, R. D. Andrews, A. M. Bishop, P. L. Boveng, D. P. Costa, D. E. Crocker, M. Haulena, M. Hindell, A. G. Hindle, R. R. Holser, S. K. Hooker, L. A. Hückstädt, S. Johnson, M.-A. Lea, B. I. McDonald, C. R. McMahon, P. W. Robinson, R. L. Sattler, C. R. Shuert, S. M. Steingass, D. Thompson, P. A. Tuomi, C. L. Williams, J. N. Womble, Best practice recommendations for the use of external telemetry devices on pinnipeds. *Anim. Biotelemetry* **7**, 20 (2019).

33. E. E. Seney, B. M. Higgins, A. M. Landry, Satellite transmitter attachment techniques for small juvenile sea turtles. *J. Exp. Mar. Biol. Ecol.* **384**, 61–67 (2010).

34. C. J. Bridger, R. K. Booth, The effects of biotelemetry transmitter presence and attachment procedures on fish physiology and behavior. *Rev. Fish. Sci.* **11**, 13–34 (2010).

35. T. Dong, C. Barbosa, Capacitance variation induced by microfluidic two-phase flow across insulated interdigital electrodes in lab-on-chip devices. *Sensors (Basel)* **15**, 2694–2708 (2015).

36. Y. Yang, J. Lu, B. D. Pflugrath, H. Li, J. J. Martinez, S. Regmi, B. Wu, J. Xiao, Z. D. Deng, Lab-on-a-fish: Wireless, miniaturized, fully integrated, implantable biotelemetric tag for real-time in vivo monitoring of aquatic animals. *IEEE Internet Things J.* **9**, 10751–10762 (2022).

37. S.-H. Lu, Y. Li, X. Wang, Soft, flexible conductivity sensors for ocean salinity monitoring. *J. Mater. Chem. B* **11**, 7334–7343 (2023).

38. J. Lu, Z. D. Deng, H. Li, M. J. Myjak, J. J. Martinez, J. Xiao, R. S. Brown, S. S. Cartmell, A small long-life acoustic transmitter for studying the behavior of aquatic animals. *Rev. Sci. Instrum.* **87**, 114902 (2016).

39. J. Martinez, T. Fu, X. Li, H. Hou, J. Wang, M. B. Eppard, Z. D. Deng, A large dataset of detection and submeter-accurate 3-D trajectories of juvenile Chinook salmon. *Sci. Data* **8**, 211 (2021).

40. S.-H. Lu, M. Samandari, C. Li, H. Li, D. Song, Y. Zhang, A. Tamayol, X. Wang, Multimodal sensing and therapeutic systems for wound healing and management: A review. *Sens. Actuators Rep.* **4**, 100075 (2022).

41. Y. Li, A. Villada, S.-H. Lu, H. Sun, J. Xiao, X. Wang, Soft, flexible pressure sensors for pressure monitoring under large hydrostatic pressure and harsh ocean environments. *Soft Matter* **19**, 5772–5780 (2023).

42. H. Li, K. W. Jung, Z. D. Deng, Piezoelectric transducer design for a miniaturized injectable acoustic transmitter. *Smart Mater. Struct.* **24**, 115010 (2015).

43. H. Li, Z. D. Deng, Y. Yuan, T. J. Carlson, Design parameters of a miniaturized piezoelectric underwater acoustic transmitter. *Sensors* **12**, 9098–9109 (2012).

44. H. Li, Z. D. Deng, J. Lu, J. J. Martinez, B. Wu, X. Zang, A new miniaturized acoustic transmitter for marine animal tracking. *IEEE J. Ocean. Eng.* **48**, 988–996 (2023).

45. M. A. Ainslie, J. G. McColm, A simplified formula for viscous and chemical absorption in sea water. *J. Acoust. Soc. Am.* **103**, 1671–1672 (1998).

46. J. M. Ingraham, Z. D. Deng, J. J. Martinez, B. A. Trumbo, R. P. Mueller, M. A. Weiland, Feasibility of tracking fish with acoustic transmitters in the Ice Harbor Dam tailrace. *Sci. Rep.* **4**, 4090 (2014).

47. Z. Li, M. Chitre, M. Stojanovic, Underwater acoustic communications. *Nat. Rev. Electr. Eng.* **2**, 83–95 (2025).

48. Z. Deng, M. Weiland, T. Carlson, M. B. Eppard, Design and instrumentation of a measurement and calibration system for an acoustic telemetry system. *Sensors* **10**, 3090–3099 (2010).

49. D. A. Righton, K. H. Andersen, F. Neat, V. Thorsteinsson, P. Steingrund, H. Svedäng, K. Michalsen, H. H. Hinrichsen, V. Bendall, S. Neuenfeldt, P. Wright, P. Jonsson, G. Huse, J. van der Kooij, H. Mosegaard, K. Hüssy, J. Metcalfe, Thermal niche of Atlantic cod Gadus morhua: Limits, tolerance and optima. *Mar. Ecol. Prog. Ser.* **420**, 1–13 (2010).

50. H. A. Broadbent, T. P. Ketterl, A. M. Silverman, J. J. Torres, Development of a CTD biotag: Challenges and pitfalls. *Deep-Sea Res. II Top. Stud. Oceanogr.* **88-89**, 131–136 (2013).

51. X. Wu, Y. Khan, J. Ting, J. Zhu, S. Ono, X. Zhang, S. Du, J. W. Evans, C. Lu, A. C. Arias, Large-area fabrication of high-performance flexible and wearable pressure sensors. *Adv. Electron. Mater.* **6**, 1901310 (2020).

52. B. You, Y. Yue, M. Sun, J. Li, D. Jia, Design of a real-time salinity detection system for water injection wells based on fuzzy control. *Sensors (Basel)* **21**, 3086 (2021).

53. B. Wu, H. Ouro-Koura, S.-H. Lu, H. Li, X. Wang, J. Xiao, Z. D. Deng, Functional materials for powering and implementing next-generation miniature sensors. *Mater. Today* **69**, 333–354 (2023).

54. N. Hammerschlag, A. J. Gallagher, D. M. Lazarre, A review of shark satellite tagging studies. *J. Exp. Mar. Biol. Ecol.* **398**, 1–8 (2011).

55. Z. A. Wang, H. Moustahfid, A. V. Mueller, A. P. M. Michel, M. Mowlem, B. T. Glazer, T. A. Mooney, W. Michaels, J. S. McQuillan, J. C. Robidart, J. Churchill, M. Sourisseau, A. Daniel, A. Schaap, S. Monk, K. Friedman, P. Brehmer, Advancing observation of ocean biogeochemistry, biology, and ecosystems with cost-effective in situ sensing technologies. *Front. Mar. Sci.* **6**, 519 (2019).

56. A. González-Santana, M. Oosterbaan, T. Clavelle, G. Maze, G. Notarstefano, N. Poffa, P. Vélez-Belchí, Analysis of the global shipping traffic for the feasibility of a structural recovery program of Argo floats. *Front. Mar. Sci.* **10**, 1161580 (2023).

57. Y. Li, G. Wu, G. Song, S.-H. Lu, Z. Wang, H. Sun, Y. Zhang, X. Wang, Soft, pressure-tolerant, flexible electronic sensors for sensing under harsh environments. *ACS Sens.* **7**, 2400–2409 (2022).

58. G. L. Lacroix, D. Knox, P. McCurdy, Effects of implanted dummy acoustic transmitters on juvenile Atlantic salmon. *Trans. Am. Fish. Soc.* **133**, 211–220 (2004).

59. C. M. Chittenden, K. G. Butterworth, K. F. Cubitt, M. C. Jacobs, A. Ladouceur, D. W. Welch, R. S. McKinley, Maximum tag to body size ratios for an endangered coho salmon (O. kisutch) stock based on physiology and performance. *Environ. Biol. Fishes* **84**, 129–140 (2008).

60. R. S. Brown, R. A. Harnish, K. M. Carter, J. W. Boyd, K. A. Deters, M. B. Eppard, An evaluation of the maximum tag burden for implantation of acoustic transmitters in juvenile Chinook salmon. *N. Am. J. Fish Manag.* **30**, 499–505 (2010).

61. M. Atkinson, C. Bingman, Elemental composition of commercial seasalts. *J. Aquaricult. Aquat. Sci.* **8**, 39–43 (1997).

62. S. T. Lebow, D. O. Foster, P. K. Lebow, Release of copper, chromium, and arsenic from treated southern pine exposed in seawater and freshwater. *For. Prod. J.* **49**, 80–89 (1999).

63. E. Lewis, The practical salinity scale 1978 and its antecedents. *IEEE J. Ocean. Eng.* **5**, 3–8 (1980).

64. J. Morison, R. Kwok, C. Peralta-Ferriz, M. Alkire, I. Rigor, R. Andersen, M. Steele, Changing Arctic Ocean freshwater pathways. *Nature* **481**, 66–70 (2012).

65. Z. D. Deng, J. J. Martinez, H. Li, R. A. Harnish, C. M. Woodley, J. A. Hughes, X. Li, T. Fu, J. Lu, G. A. McMichael, M. A. Weiland, M. B. Eppard, J. R. Skalski, R. L. Townsend, Comparing the survival rate of juvenile Chinook salmon migrating through hydropower systems using injectable and surgical acoustic transmitters. *Sci. Rep.* **7**, 42999 (2017).

66. Y. Yang, R. Elsinghorst, J. J. Martinez, H. Hou, J. Lu, Z. D. Deng, A real-time underwater acoustic telemetry receiver with edge computing for studying fish behavior and environmental sensing. *IEEE Internet Things J.* **9**, 17821–17831 (2022).

67. Connector care and cable installation. Application Note No. 57 (Sea-Bird Electronics, Inc., 2014).

68. M. A. Weiland, Z. D. Deng, T. A. Seim, B. L. LaMarche, E. Y. Choi, T. Fu, T. J. Carlson, A. I. Thronas, M. B. Eppard, A cabled acoustic telemetry system for detecting and tracking juvenile salmon: Part 1. Engineering design and instrumentation. *Sensors (Basel)* **11**, 5645–5660 (2011).

69. Z. Wang, L. Goodman, Evolution of the spatial structure of a thin phytoplankton layer into a turbulent field. *Mar. Ecol. Prog. Ser.* **374**, 57–74 (2009).

70. L. Goodman, Z. Wang, Turbulence observations in the northern bight of Monterey Bay from a small AUV. *J. Mar. Syst.* **77**, 441–458 (2009).

71. A. J. Read, “Biotelemetry,” in *Encyclopedia of Marine Mammals*, B. Würsig, J. G. M. Thewissen, K. M. Kovacs, Eds. (Academic Press, 3rd Ed., 2018), pp. 103–106.

72. A. M. Treasure, F. Roquet, I. J. Ansorge, M. N. Bester, L. Boehme, H. Bornemann, J.-B. Charrassin, D. Chevallier, D. P. Costa, M. A. Fedak, C. Guinet, M. O. Hammill, R. G. Harcourt, M. A. Hindell, K. M. Kovacs, M.-A. Lea, P. Lovell, A. D. Lowther, C. Lydersen, T. McIntyre, C. R. McMahon, M. M. C. Muelbert, K. Nicholls, B. Picard, G. Reverdin, A. W. Trites, G. D. Williams, P. J. N. de Bruyn, Marine mammals exploring the oceans pole to pole: A review of the MEOP consortium. *Oceanography* **30**, 132–138 (2017).

73. H. A. Broadbent, T. P. Ketterl, C. S. Reid, J. Dlutowski, A low-cost, miniature CTD for animal-borne ocean measurements, in *OCEANS 2010 MTS/IEEE SEATTLE* (2010), pp. 1–7.

74. H. A. Broadbent, *A CTD Biotag for Mid-Sized Marine Predators* (University of South Florida, 2012).
